# Integrated thermal and phytoremediation of agricultural soils impacted by PFAS

**DOI:** 10.1073/pnas.2600786123

**Published:** 2026-07-21

**Authors:** Jake T. Thompson, Millie Dobson, Tim Jesper Suhrhoff, Yoshiki Kanzaki, Chloe Kent, Lucinda Bryce, Ella Milliken, Christopher T. Reinhard, Yuan Yao, Noah Planavsky

**Affiliations:** ^a^ Department of Earth and Planetary Sciences, Yale University, New Haven, CT 06511; ^b^ School of Ocean and Earth Science, National Oceanography Centre Southampton, University of Southampton Waterfront Campus, Southampton SO14 3ZH, United Kingdom; ^c^ Yale Center for Natural Carbon Capture, Yale University, New Haven, CT 06511; ^d^ School of Earth and Atmospheric Sciences, Georgia Institute of Technology, Atlanta, GA 30332; ^e^ Department of Earth, Environmental, and Planetary Sciences, Brown University, Providence, RI 02912; ^f^ Center for Industrial Ecology, Yale School of the Environment, Yale University, New Haven, CT 06514

**Keywords:** enhanced weathering, biochar, PFOA, PFOS, plant-uptake

## Abstract

Per- and polyfluoroalkyl substances (PFAS) are persistent synthetic compounds that have contaminated millions of hectares of agricultural land through decades of biosolids application. Conventional remediation approaches, such as thermal destruction or excavation, are prohibitively expensive, carbon intensive, and leave affected farmland unfit for agriculture. Here, we present a potential scalable remediation strategy that combines phytoremediation, biochar production, and enhanced weathering to simultaneously remove PFAS from soil, immobilize residual contamination, and achieve durable carbon dioxide removal (CDR). Using stochastic modeling constrained by experimental data, we show that soil pH management through alkaline rock amendment can accelerate PFOS removal, shortening remediation timelines by more than a decade under typical contamination levels. Pyrolysis of harvested biomass effectively destroys PFAS and produces biochar, which, when reapplied to soil, substantially reduces leaching to groundwater and the surrounding environment. National-scale simulations across the estimated one million hectares of PFAS-impacted cropland indicate a combined CDR potential of approximately 11 Mt CO_2_ y^–1^, equivalent to 4 to 6% of the US 2050 carbon removal target. We estimate a median remediation cost of $1,460 USD ha^–1^ y^–1^—more than an order of magnitude lower than current technologies, with costs substantially reduced through carbon removal revenues valued near the social cost of carbon. This integrated thermal and phytoremediation framework provides a viable pathway to restore contaminated farmland, mitigate PFAS exposure risks, and contribute meaningfully to national climate mitigation goals.

## Significance

Widespread biosolids application has introduced per- and polyfluoroalkyl substances (PFAS) into millions of hectares of agricultural soils, yet existing remediation methods are costly, carbon intensive, and impractical at scale. We evaluate an integrated strategy that combines phytoremediation, biomass pyrolysis, and enhanced weathering to remove PFAS while generating durable carbon dioxide removal (CDR). Using stochastic modeling constrained by experimental data, we show that raising soil pH with alkaline rock amendments increases PFAS mobility and plant uptake, shortening remediation timelines by more than a decade under typical contamination levels. National-scale simulations yield a combined CDR potential of ~10.5 Mt CO_2_ y^–1^. With remediation costs decreased by an order of magnitude lower compared to conventional approaches.

Per- and polyfluoroalkyl substances (PFAS) are a large class of synthetic chemicals which have become ubiquitous in consumer and industrial applications (1, 2). Their persistence in the environment has earned them the label “forever chemicals.” Nearly all people now have detectable levels of PFAS in their blood (3–5), and exposure has been linked to cancer, liver damage, thyroid disease, immune dysfunction, and reproductive disorders, among other health issues (6, 7). Because PFAS resist natural degradation, they accumulate in soils and water once released, posing long-term risks to human health and ecosystems (8, 9).

Decades of widespread PFAS use have led to their release into wastewater (10), where they concentrate in sewage sludges, also known as biosolids (8, 11, 12). These biosolids, often applied to farmland as a nutrient-rich fertilizer, represent a major pathway for PFAS to enter agricultural soils, groundwater, and the food system. This is a nearly worldwide problem, but one that has recently emerged as a key policy priority in the United States. A recent US Environmental Protection Agency risk assessment showed that perfluorooctane sulfonic acid (PFOS) and perfluorooctanoic acid (PFOA) in biosolids can exceed human health thresholds at typical biosolid application rates (13). States such as Maine have already shut down farms where PFAS levels surpassed regulatory limits, highlighting the growing challenge these contaminants pose for agricultural communities (14, 15).

Remediating PFAS-contaminated farmland is currently infeasible at scale. Established methods—thermal destruction or excavation and landfilling—cost an estimated $0.8 to 1.6 million per hectare (16). With millions of hectares of US farmland likely affected by biosolids use (17), remediation costs could run into the trillions of dollars using traditional approaches. Beyond cost, these methods strip away topsoil and release large quantities of carbon dioxide. A scalable, sustainable, and cost-effective approach toward remediating PFAS-contaminated farmland is urgently needed.

Here, we present and evaluate the feasibility, impacts, and cost of a remediation strategy that integrates phytoremediation paired with soil pH management and biochar production, building on growing interest in natural solutions that link remediation with carbon dioxide removal (CDR) (18). This combined approach aims to accelerate PFAS removal from soil, immobilize residual contamination, and restore farmland while simultaneously achieving durable CDR. We provide the first quantitative assessment of the remediation potential, costs, and scalability of this strategy across US croplands, offering a potential path toward addressing a pressing environmental challenge over the coming decades.

## Materials and Methods

Here we provide a high-level summary of the methods used to estimate the scale of PFAS-impacted agricultural land, model integrated remediation outcomes, and compare this approach with conventional remediation scenarios. We estimated the extent of PFAS-impacted agricultural land in the United States by combining historical biosolids generation and land-application records with literature-based agronomic application rates. PFAS concentration distributions were parameterized using the Maine Department of Environmental Protection PFAS site assessment dataset, filtered to focus on sludge-utilization sites representative of agricultural biosolids application. Impacted land was spatially allocated across states using biosolids production estimates, agricultural land area, and population-weighted placement of 1,000-ha grid cells. Baseline site conditions, including soil PFAS concentrations, pH, and organic carbon content, were then used to initialize remediation and CDR scenarios.

We modeled an integrated remediation strategy combining phytoremediation, soil pH management, biochar amendment, enhanced weathering, and pyrolysis of harvested biomass. Plant uptake of PFOS and PFOA was estimated using pH-dependent sorption relationships, distribution coefficients, and published plant-removal efficiencies for hemp and red fescue, with uncertainty propagated through Monte Carlo simulations. Biochar amendment was modeled as a sorption-based strategy to reduce residual PFAS bioavailability, while enhanced weathering CDR was estimated using SCEPTER simulations. Biochar carbon storage was calculated from spatially variable biomass productivity, pyrolysis yield, transport emissions, and process emissions. Conventional remediation scenarios, including hazardous-waste landfilling and rotary kiln treatment, were modeled for comparison using literature-derived cost, transport, and emissions parameters. Detailed methodology for each specific analysis is provided in *SI Appendix*, sections 1.1 to 1.18.

### Remediation Pathway.

Our proposed remediation method operates in two stages. First, the application of alkaline rock amendments to soil (or enhanced weathering) elevates soil pH to a target pH of 7, leading to an increase in mobility (19) and bioavailability (20) of key PFAS such as perfluorooctane sulfonate (PFOS) and perfluorooctanoic acid (PFOA), which are the primary focus of this analysis due to their regulatory relevance in agricultural soils. Next, the PFAS-containing hemp biomass is pyrolyzed under high-temperature conditions (typically >500 to 700 °C) with downstream oxidation of off-gases (>900 °C), ranges shown to remove and destroy targeted PFAS (21–23). This has been shown to produce biochar which is free of targeted PFAS (24). Although a complete fluorine mass balance during thermal treatment has not yet been established (25), the PFAS concentrations typical of agricultural biomass correspond to extremely small total fluorine inventories, such that potential byproducts (e.g., HF or fluorocarbons) would occur at very low absolute mass (*SI Appendix*, section 17).

The biochar can be reapplied to soils or transported to sites where PFAS contamination is too high to be addressed with phytoremediation alone. Biochar has been shown to be effective at immobilizing PFOS, able to achieve 99% reduction in leaching in soils at application rates as low as 1 to 5% by mass (26). The biochar therefore provides a means to limit PFAS transfer into well waters. However, the long-term stability of PFAS immobilization by biochar under field conditions remains uncertain, as aging, shifts in soil chemistry, and competitive sorption with natural organic matter may reduce sorption capacity over time (27). With continued application, the ratio of biochar to contaminated soil increases, further reducing PFAS bioavailability by competitive sorption (9, 26, 28). In addition to remediating soils, both addition of biochar to soils (29) and enhanced weathering (30) are promising nature-based approaches to remove carbon dioxide from the atmosphere and reduce greenhouse gas emissions from crop systems such that this pathway represents a multifunctional approach where environmental remediation and climate change mitigation can be addressed synergistically and be used to potentially cross-finance each other. The phytoremediation and biochar components are evaluated using a data-constrained modeling framework that couples pH-dependent PFAS partitioning, experimentally derived plant uptake relationships, and stochastic simulations to gauge uncertainty (see *SI Appendix* for details).

This dual extraction and immobilization approach allows for a significant degree of flexibility—at sites where PFAS concentrations are relatively low and regulatory thresholds are within reach; enhanced weathering can accelerate phytoremediation to meet soil screening levels. In contrast, biochar amendment can be used to immobilize PFAS and mitigate key exposure risks at sites where phytoremediation alone cannot meet soil thresholds and there is a clear risk of groundwater contamination.

**Fig. 1.**
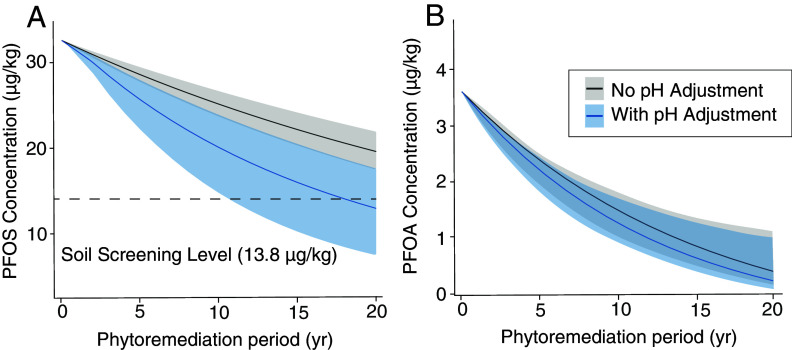
Modeled PFOS and PFOA concentrations in soil under proposed remediation strategy. Modeled trajectories of plant-driven PFOS and PFOA removal in soils with and without a pH amendment. (*A*) Extractable PFOS concentrations in soil with the relevant agricultural screening level via the pasture–milk pathway (14) and (*B*) Extractable PFOA concentrations in soil. In each panel, the solid line represents the modeled outcome under average site conditions, and the shaded region denotes the 95% CI.

Our modeling shows that PFAS concentrations in agricultural soils can potentially be reduced to below current risk-based thresholds for agricultural soils in the United States within decade scales in most regions (Fig. 1*A*). However, soil chemistry plays a critical role in remediation rates. Managing soil pH through enhanced weathering accelerated PFOS decline by 20 to 40%, cutting remediation timelines by more than a decade at typical contamination levels. This effect is less pronounced for PFOA (Fig. 1*B*). However, for PFOS, one of the compounds of primary regulatory concern, our modeling strongly suggests that pH management substantially improves outcomes (Fig. 1*A*).

The remediation process is governed by the soil–water partitioning behavior of PFAS, often described by the distribution coefficient (K_d_). Importantly, K_d_ values are inversely correlated with plant uptake (20). When K_d_ values are lower, PFOS remains more mobile and bioavailable to plants (19, 20). This relationship can be deliberately manipulated, although this response is mediated by multiple interacting soil properties including organic carbon content, mineralogy, and solution chemistry. Raising soil pH via alkaline rock amendments lowers K_d_, thereby enhancing plant uptake and accelerating phytoremediation (Fig. 1*A*), although this response will be site specific since K_d_ is mediated by multiple interacting soil properties (31). Additionally, lowering K_d_ increases PFAS mobility and may temporarily elevate leaching potential. This managed mobilization facilitates phytoextraction and reduces the total PFAS mass remaining in soils over time, thereby reducing the mass of PFAS entering surface water and groundwater systems. In contrast, adding treatment-derived biochar increases K_d_ through strong PFOS sorption, suppressing plant uptake while substantially reducing the risk of leaching to groundwater. Although this decreases phytoextraction, it is advantageous when groundwater protection is the primary concern. In practice, the approach can be tuned to balance retention and uptake depending on site-specific management priorities, particularly where groundwater protection is a primary concern.

Through phytoremediation, PFOS concentrations in soil exponentially decline as hyperaccumulating crops (e.g., hemp and perennial grasses) draw down the contaminant pool. Harvested biomass converted to biochar and reapplied to the field can also serve to immobilize residual PFAS, thereby reducing their transfer into forage crops. This biochar pathway is particularly valuable at highly contaminated sites (>100 µg/kg PFOS). Under scenarios with elevated contamination (e.g., the highest 90th percentile of sites), the combination of phytoremediation and transition to biochar addition reduced forage crop concentrations below conservative food-chain thresholds (32) within a decade—an outcome not achievable with phytoremediation alone (*SI Appendix*, section 1.6–1.9). At sites with lower pollution levels, it may be more effective to not add biochar such that higher rates of PFAS phytoremediation can be sustained until remediation below critical levels is complete. These results demonstrate that agricultural soils can be shifted from PFAS reservoirs into actively managed systems with reduced long-term risk. In addition, unlike traditional PFAS remediation approaches, this strategy empowers the communities most impacted by contamination—farmers—to directly remediate their land while potentially maintaining productive use.

We estimate that between 0.6 and 2.4 million hectares of US cropland have been strongly impacted by PFAS over the past five decades (*SI Appendix*, Figs. S3–S5), with a likely median value of roughly 1 million hectares. This estimate is conservative, as it excludes applications prior to 1976 and does not account for additional inputs such as wastewater effluent irrigation (10, 33), paper mill sludge (34), or PFAS-containing pesticides (35, 36). We estimate a mean soil burden of ~70 ng g^–1^ ∑_21_ PFAS, with PFOS emerging as the dominant compound (average 35 ng g^–1^). These levels are consistent with reported ranges at biosolids-impacted sites globally and exceed proposed regulatory thresholds in several US states (14, 37, 38), making PFOS the primary constraint on safe agricultural use on millions of arable acres. Without adoption of remediation strategies such as the one proposed here, PFAS impacted land will continue to pose risks to food security, ecosystem health, and groundwater quality.

### CDR Potential.

**Fig. 2.**
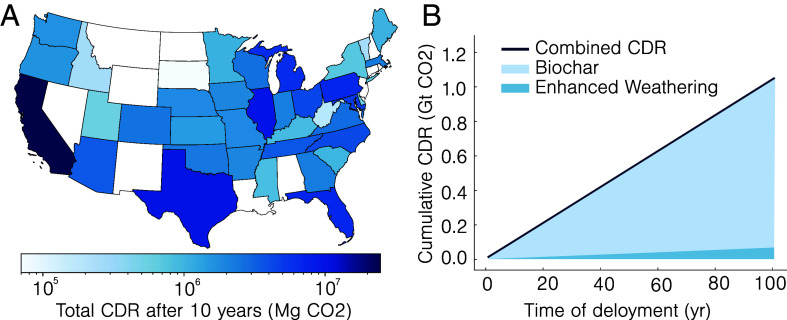
CDR potential from biochar and EW on PFAS-impacted farmland in the US. (*A*) State-level CDR potential across impacted agricultural areas. (*B*) Cumulative national CDR over time showing contributions from biochar and EW.

The remediation strategy for PFAS-contaminated agricultural soils proposed here offers both an approach toward pollution management and a meaningful contribution to climate mitigation. We estimate a combined median national CDR potential in the United States of ~10.5 Mt CO_2_ y^–1^ through this approach, with enhanced weathering contributing ~0.6 ± 0.2 Mt CO_2_ y^–1^ and biochar production ~9.9 ± 3.7 Mt CO_2_ y^–1^ (Fig. 2). These estimates reflect CO_2_ only (not CO_2_-equivalent) and are subject to uncertainty from several sources, including the total extent and spatial distribution of PFAS-impacted land, variability in biomass productivity and biochar yield, assumptions around pyrolysis emissions and transport distances, and the treatment of long-term carbon stability in biochar (See SI). Nonetheless, the dominant source of uncertainty is the total area of PFAS-impacted land available for deployment, indicating the need for additional sampling campaigns.

To place this in context, US CDR targets for achieving net-zero by 2050 are on the order of 0.2 Gt CO_2_ y^–1^ (39, 40); thus, adoption of this strategy could achieve approximately 4 to 6% of the national goal. Over a 20-y remediation period, this equates to roughly 210 Mt CO_2_ removed. In contrast, conventional remediation approaches such as excavation with landfill disposal or thermal treatment of the top 30 cm of soil are associated with substantial greenhouse gas emissions, ranging from 92 to 800 tCO_2_ ha^–1^ and totaling 110 to 960 Mt CO_2_ across the same land area over the same timeframe (*SI Appendix*, section 1.13 and Fig. S7). Together, this strategy, relative to conventional remediation pathways, avoids the emissions of hundreds of megatons of CO_2_.

Enhanced weathering estimates are based on applying finely ground basalt until soils reach a target pH of 7 and adding material annually to maintain this target, using a reactive transport model designed to mimic weathering in croplands (41, 42). We assume an 80% effective efficiency from the model estimates, reflecting ~10% loss of alkalinity during river–ocean transport, and ~10% life cycle and logistics emissions consistent with prior enhanced weathering studies (43) (*SI Appendix*, section 1.10 and 1.11). Basalt was selected for its favorable CDR potential, nutrient content, and relatively low heavy metal risk. However, limestone may be more favorable in many regions and can also drive carbon removal (44). Any amendment selection should consider local soil chemistry and trace metal content to avoid unintended cocontaminant inputs. Although additional region- and process-specific Life Cycle Assessments (LCAs) are needed for comprehensive analysis of all GHG emissions and sinks associated with the proposed strategies, these results provide a first-order estimation for the feasibility and climate cobenefits of this remediation pathway.

### Economic Feasibility.

The proposed phytoremediation–biochar–enhanced weathering system delivers PFAS remediation at far lower cost than conventional technologies. Without carbon crediting, an estimated conservative mean remediation cost using this approach is approximately 4,200 ± 650 USD ha^–1^ y^–1^ (all costs reported in US dollars—*SI Appendix*, sections 1.14 to 1.16), with the main driver of cost being the transport and pyrolysis of harvested biomass (*SI Appendix*, Fig. S9). However, incorporating a social cost of carbon (SCC) of 190 USD tCO_2_^–1^ offsets roughly 70% of this total, reducing net remediation expenditures to ~1,460 ± 700 USD ha^–1^ y^–1^.

Beyond the base cost of deploying this strategy we also assume a farmer incentive of 300 to 500 USD ha^–1^ y^–1^, based on approximate averages of net cash farm income for US crop production businesses (45). This would allow for maintaining agricultural productivity and provide farmers with a stable income stream. If CDR were valued at 275 USD tCO_2_^–1^, within the range for current durable carbon credits (46, 47), carbon revenues would fully cover all remediation costs (*SI Appendix*, Fig. S10). These SCC values are consistent with recent economic analyses, which place the social cost of carbon in the ~190 to 300 USD tCO_2_^–1^ range (47). We are not advocating for a purely carbon market-based solution for agricultural PFAS remediation. We are simply highlighting that our analysis suggests the potential income from durable carbon sequestration from this process could more than cover typical farmer profits in row crops and even the cost of remediation.

By contrast, conventional soil remediation methods, typically applied to highly localized and heavily contaminated sites, remain prohibitively expensive. Thermal treatment and excavation with off-site disposal cost an estimated 0.8 to 1.6 million USD per hectare (16), which is substantially higher than the estimated 29,000 USD per hectare for our strategy over a 20-y remediation period. Applied across the approximately 1.2 million hectares of PFAS-contaminated farmland, these conventional approaches would translate to a total cost of 1.3 to 2.4 trillion USD (*SI Appendix*, Fig. S11). In addition to the financial burden, which is 30 to 50 times higher than our proposed system, these approaches also permanently remove agricultural land from productive use due to the removal or destruction of topsoil, creating further economic stress for affected communities as they are forced to transition their land away from agriculture.

Biochar expenditures were estimated using a cost-based model, incorporating capital cost, transport, and processing operations as well as preprocessing, storage, and residual handling (*SI Appendix*, section 1.14). Median production costs were 161 USD tCO_2_^–1^ for large centralized facilities and 156 USD tCO_2_^–1^ for mobile units (*SI Appendix*, Fig. S9). Capital costs were 15% of the total project costs. ERW deployment costs were taken from established literature estimates of approximately 160 USD tCO_2_^–1^ (43). However, the dominant cost driver for this system is the production and management of hemp and fescue biomass, which determines both phytoremediation capacity and the quantity of material requiring processing. This component also has the greatest potential for cost reduction through selective breeding, genetic modification, and the discovery of new hyperaccumulator species such as the recent *Oenothera rosea*, which exhibits PFOS remediation rates nearly twice those of the hemp used here (48, 49). If commercialized and deployed at scale, such improved phytoremediators could substantially accelerate PFAS extraction and reduce total system costs. Future research should conduct region-specific and process-explicit techno-economic analysis (TEA) to explore different pyrolysis biorefinery configurations, supply chain design, and financial structures for real-world implementation.

**Fig. 3.**
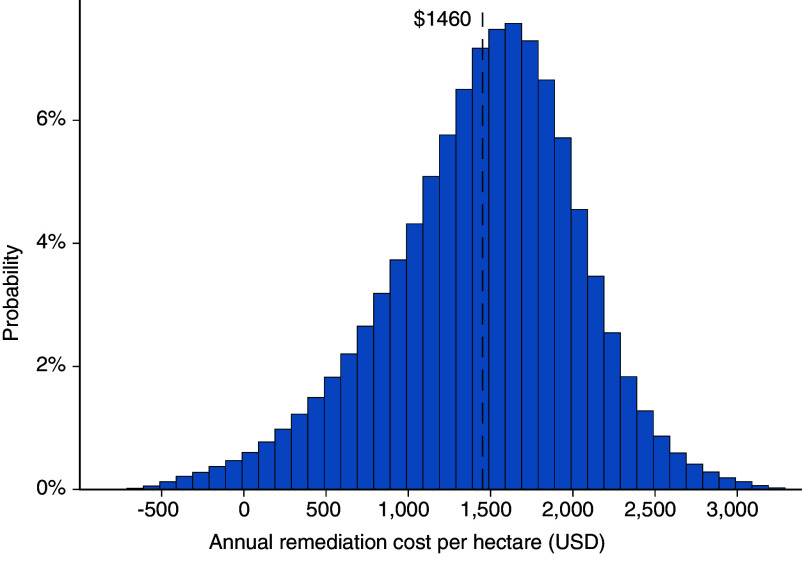
Distribution of annual remediation costs. Histogram shows the distribution of annual remediation costs generated by sampling biomass production costs, carbon removal rates, credit prices at $190 USD, treatment costs, and farmer income offsets from their empirical ranges. The dashed line indicates the median annual remediation cost.

The combined thermal and phytoremediation strategy proposed here leverages existing agricultural infrastructure, equipment, and workforces rather than the limited capacity and transport of soil burners or excavation equipment, a large reason for the stark reduction in cost. As a result, deployment could be brought to scale relatively rapidly. Importantly, this approach provides landowners with autonomy over the remediation process, in many cases enabling them to farm their land in a relatively familiar way while preserving agricultural value, generating a potential income stream, and allowing eventual transition back to traditional cash crop production (Fig. 3).

### Conclusion.

PFAS contamination of agricultural soils represents a pervasive and persistent environmental challenge, with legacy biosolids applications having impacted millions of hectares of US farmland and farms worldwide. In the US soil PFAS concentrations frequently exceed proposed regulatory thresholds and limits for safe agricultural use. Current remediation methods, including thermal destruction and excavation with landfilling, are economically and logistically infeasible at scale, threatening farmland productivity while generating hundreds of millions of tons of CO_2_ emissions.

We propose a remediation strategy combining phytoremediation, biochar production, and enhanced weathering. Our initial feasibility analysis suggests that this pathway could offer a scalable, cost-effective alternative to traditional soil remediation pathways. Soil pH management accelerates PFOS removal, cutting remediation timelines by more than a decade at typical contamination levels. At sites with particularly high contamination, biochar immobilization reduces leaching to groundwater by more than 95% and can bring agricultural plant concentration to below agricultural risk thresholds within roughly a decade in the vast majority of sites. This dual approach allows flexibility on how each site is managed depending on site characteristics. The approach also provides significant carbon benefits—potentially providing 4 to 6% of US CDR targets in 2050 for 1.5 °C of warming (40), demonstrating the dual environmental benefits of soil remediation and durable carbon removal. Economically, the strategy reduces remediation costs by over an order of magnitude relative to conventional methods, making it one of the lowest-cost approaches proposed for addressing PFAS contamination in croplands.

Although this analysis is centered on US cropland, PFAS contamination from biosolid application is a global issue. In Europe, approximately 50% of the 10 million tons of dry sewage sludge generated annually is applied to agricultural land (50), with the U.K. spreading ~70% (~1 million tons) of its biosolids (51). China applies ~5 million dry tons of biosolids to soils each year (52), while in Australia, 79.3% of the 372,000 tons produced in 2023 were used in agriculture (53). Given the persistent PFAS content in these materials, the remediation framework presented here is broadly applicable, providing a model for sustainable PFAS management worldwide.

## Supplementary Material

Appendix 01 (PDF)

## Data Availability

All study data are included in the article and/or *SI Appendix*.
