# Supplementary material for: Integrated thermal and phytoremediation of agricultural soils impacted by PFAS
Source: Proc Natl Acad Sci U S A. 2026 Jul 21;123(30):e2600786123. doi: 10.1073/pnas.2600786123 (PMC13416631; doi:10.1073/pnas.2600786123)
Supplement: Supplementary file 1 — Appendix 01 (PDF) [file pnas.2600786123.sapp.pdf]

Supplementary Materials for

Integrated Thermal and Phyto-Remediation of Agricultural Soils Impacted by PFAS

Authors: Jake T Thompson[1], Millie Dobson[2], Tim Jesper Suhrhoff[3], Yoshiki Kanzaki[4], Chloe Kent[1], Lucinda Bryce[5], Ella Milliken[1], Christopher T Reinhard[4], Yuan Yao[6], Noah Planavsky[1]*

Affiliations:

[1]Department of Earth and Planetary Sciences, Yale University, 10 Whitney Ave., New Haven CT 06511 USA

[2]School of Ocean and Earth Science, National Oceanography Centre Southampton, University of Southampton Waterfront Campus, Southampton SO14 3ZH, UK

[3]Yale Center for Natural Carbon Capture, Yale University, New Haven, Connecticut 06511, USA

[4]School of Earth & Atmospheric Sciences, Georgia Institute of Technology, Atlanta, Georgia 30332, USA

[5]Department of Earth, Environmental, and Planetary Sciences, Brown University, Providence, Rhode Island 02912, USA

[6]Center for Industrial Ecology, Yale School of the Environment, Yale University, New Haven. CT 06514, United States of America

* corresponding author: noah.planavsky@yale.edu

Contents

1. Materials and Methods ... 3
1.1 Estimated mass of biosolids land applied in the U.S. ... 3
1.2 Area of impacted land ... 3
1.3 PFAS concentration and frequency ... 4
1.4 Spatial Location of PFAS Impacted Land ... 5
1.5 Remediation site conditions ... 6
1.6 Modeling plant uptake efficiency based on Kd and pH change ... 7
1.7 Phytoremediation ... 8
1.8 Biochar Amendment and Sorption Coefficients ... 10
1.9 Risk based thresholds for plant concentrations ... 12
1.10 Enhanced weathering CDR estimates ... 13
1.11 Transport of ERW feedstock estimates ... 14
1.12 Biochar CDR Estimation ... 18
1.13 Traditional remediation emission estimates and costs ... 22
1.14 Cost of Biochar Production ... 25
1.15 Remediation cost estimates ... 32
1.16 Breakeven cost scenarios ... 33
1.17 Pyrolysis Emmissions and Infrastructure Requirements for Biomass Processing ... 34
1.18 Model Limitations and Future Work ... 38
2.0 FIGURES ... 40
3.0 TABLES ... 51

1. Materials and Methods

1.1 Estimated mass of biosolids land applied in the U.S.

The mass of biosolids generated and land-applied in the U.S. was derived from two primary data sources: (1) a national inventory of biosolids generation and application (*1*) and (2) the National Biosolids Generation Project database, which contains state- and national-level inventories(*2*). These datasets provided national estimates of land-applied biosolids for the years 1976, 1984, 1988, 1998, 2004, 2010, and 2018.

To estimate the annual biosolids land application for years not explicitly reported, interpolation and extrapolation methods were applied. For years between recorded data points, the average of the two adjacent values was used as the estimated mass. For years prior to the earliest data point and post latest (e.g., 1975, 2019 and 2020) the last available datapoint was extrapolated. These values were compiled for the years 1975–2020 and summed to estimate the total mass of biosolids land-applied over this period. This range was selected based on historical evidence of PFAS in biosolids dating back to 1976(*3*) and the continued use of PFAS containing biosolids on agricultural land to the modern day(*4*).

1.2 Area of impacted land

The area of impacted agricultural land within the U.S. was estimated using standard land application rates based on literature accounts and state biosolid application recommendations (*5*–*7*). These rates ranged from 6.3 – 10.1 t hectare^{-1} year^{-1}.

$$B_h = \text{application rate} \times \text{duration of application} \quad (1)$$

With the average application rate being the same value used for EPA's draft biosolid risk assessment for PFOA and PFOS 7.6 t hectare^{-1} year^{-1}. These values fall within agronomic

estimates conducted by EPA previously (2.5 – 11 t hectare^{-1} year^{-1}) (*8*). Where much of the uncertainty comes from in this assessment is the lack of information on the amount of time biosolids were applied to land. Biosolid application permit records exist for many states however the length of time, application rate, and if biosolids were even added is rarely documented. For this assessment we have constrained the time of application to once per year for 7 to 20 years of application (parameters for all equations used in this section are in Table S1)

$$H_I = \frac{C_b}{B_h} \quad (2)$$

1.3 PFAS concentration and frequency

The concentrations of PFAS and the distribution of their detection frequency at an impacted agricultural site come from a relatively robust dataset (Maine DEPs PFAS state assessment)(*9*). At the time of this analysis this data contained ~800 datapoints for PFAS impacted sites. To refine the dataset for analysis, samples that were not categorized as "Sludge Utilization Sites" were excluded, as the dataset also included entries labeled as "Surface Spills" and "Septage Storage." Additionally, any samples containing the terms "Landfill" or "Airport" were removed to ensure that the remaining data primarily reflected farmland biosolid applications (hereafter referred to as "Farm Data"). This filtering process resulted in the removal of approximately 200 samples from the dataset.

The remaining 563 sites contained data for total $\sum_{21}$ PFAS (PFBA, PFBS, PFDA, PFDoA, PFDS, PFHpA, PFHpS, PFHxA, PFHxDA, PFHxS, PFNA, PFNS, PFOA, PFODA, PFOS, PFOSA, PFPeA, PFPeS, PFTeA, PFTriA, and PFUnDA). The average concentration of $\sum_{21}$ PFAS and the frequency of detection at that concentration was recorded as well as the average

concentration for each PFAS. The location and concentration of PFAS at these sites were plotted (Fig S1) and relative concentrations of $\sum_{21}$ PFAS across the sites (Fig S2).

1.4 Spatial Location of PFAS Impacted Land: State, Point, and Fraction of Agricultural Land

After determining the total amount of impacted land within the U.S., estimates were made to find the general distribution of this land across states. For these estimates two nationwide assessments detailing the amount of biosolids created for land application by each state were sourced (*2*, *5*). This data provided the ratio of biosolids produced by each state which was then multiplied by the total amount of impacted land within the U.S. to get an estimated amount of land within each state impacted by PFAS through biosolid application. This data was then used in comparison to a USDA database on the amount of agricultural land in farms for each state (*10*). The ratio of "land in farms" to the estimated median amount of impacted land in each state provided the data presented in Fig S3.

For CDR determinations, a higher resolution estimate of the amount of impacted land was needed. In this case the amount of impacted land from each state was divided into 1,000-hectare blocks rounded down. The blocks were then assigned a random coordinate within each states geometry provided by R's "maps" package(*11*). These points were then reselected within each state but weighted by population. This was done because wastewater treatment plant load and biosolids production are heavily influenced by population served and the cost benefit of using biosolids as an agricultural amendment is strongly impacted by transport distance(*12*). To spatially distribute these blocks within each state, latitude–longitude points were sampled with probabilities proportional to local population density coming from U.S. Census data (*13*). For each spatial unit j within state i, the selection weight was defined as:

$$Weighted\ Cordinates = \frac{D_{ij}}{\sum_j D_{ij}}\ (3)$$

where *Dij* is the population density of unit *j*. Weighted random sampling was performed for each 1,000 hectare block generated per state, with replacement, to generate point coordinates representing impacted areas. The resulting data for the 1,000 hectare plots is shown in the Fig S4 and the relative amount of agricultural land for each state in Fig S5.

1.5 Remediation site conditions

The site conditions from this baseline theoretical site comes from special mapping data from Maine DEPs remediation program (*9*). To initialize year-zero site conditions for the model, we extracted soil pH values across Maine croplands using an empirical Bayesian kriging raster dataset(*14*). These values were matched to field sample coordinates from known sludge-impacted sites. Each sample location was georeferenced and overlaid onto the raster, allowing soil pH to be spatially assigned to each site. This provided a realistic distribution of initial soil pH conditions used to simulate PFAS partitioning and remediation behavior in year zero of the model. For both soil pH and PFAS concentrations the median value across all sites (n=563) was selected.

For total organic carbon (TOC) and the fraction of organic carbon (f_{oc}), values were randomly assigned within a realistic range observed in northeastern agricultural soils. Specifically, foc was sampled from a uniform distribution between 0.01 and 0.03, and TOC was then calculated as 100 × f_{oc}. These values reflect typical soil organic carbon levels for surface horizons in cultivated areas of Maine. Since partitioning of PFAS to soil is driven in part by organic carbon content, this step was critical for estimating initial distribution coefficients (K_d) and aqueous concentrations (C_w) under year-zero conditions.

1.6 Modeling plant uptake efficiency based on Kd and pH change

To simulate PFAS uptake dynamics in plants across varying soil conditions, we constructed a modeling framework linking soil pH to PFAS sorption and subsequent plant removal efficiencies. Experimental data on PFAS sorption to soil organic fraction (K_{oc}) in high TOC soils from Campos-Pereira et al. (2023) was used to develop compound-specific linear models describing log K_{oc} as a function of pH for PFOS and PFOA.

$$\log_{10}(K_{\mathrm{oc}}) = m \cdot \mathrm{pH} + b \tag{4}$$

Each model was fit using empirical pH-log K_{oc} relationships and then used to generate predicted K_{oc} values for each compound at target pH values (e.g., 5.5, the reported pH in pot trials from (Nassazzi et al. 2023). These log K_{oc} values were back-transformed and multiplied by simulated site-specific soil organic carbon fractions to calculate partition coefficients per compound per site.

Next, PFAS uptake efficiency was modeled as a function of K_d using exponential decay models fit to observed removal efficiencies in hemp (*15*). Reported removal efficiencies for perfluoroalkyl carboxylic acids (PFCAs) and perfluoroalkyl sulfonic acids (PFSAs) were correlated with their respective K_{oc}, which were either calculated using the Campos-Pereira pH-dependent K_{oc} models(*16*) or obtained from published literature (*17–19*) . Removal efficiency data and corresponding K_{oc} values from Nassazzi et al. 2023 (*15*) were compiled into a dataset and the exponential decay model of the form:

$$R_{\mathrm{plant}} = a \cdot \exp(-b \cdot K_{\mathrm{oc}}) \tag{5}$$

was fit to the data using nonlinear least squares regression to capture the relationship between removal efficiency in hemp and K_{oc} values. Log-transforming the removal efficiency allowed linear regression to estimate model parameters a and b, which characterize the exponential decay trend. Similarly, for PFSAs, removal efficiencies were modeled as a function of K_{oc} using the same exponential approach. The distinction between PFCAs and PFSAs was made because they exhibit different sorption and plant uptake behaviors(*16*, *18*, *20*).

1.7 Phytoremediation

The phytoremediation scenario presented soil conditions and PFAS concentrations as described in the site conditions above. In short, these PFAS concentrations are the average values for the state assessment conducted in Maine for suspect PFAS impacted agricultural sites (n= 563). The presented remediation situation was selected as a realistic scenario where hemp is planted in the active growing season and harvested after ~120 days, that biomass is collected for pyrolysis and red fescue grass is planted (a cover crop shown to have high remediation potential(*21*)), next growing season the fescue is removed and bailed, and hemp is planted again to start the cycle over again.

Soil PFAS concentrations are represented as extractable concentrations, consistent with the underlying datasets used to parameterize the model. As such, modeled plant uptake is based on extractable PFAS rather than the strictly bioavailable fraction. While extractable concentrations are commonly used in regulatory frameworks to assess contamination, the bioavailable fraction represents only a subset of total PFAS and is more directly linked to plant uptake and exposure pathways. Extractable concentrations are commonly used as practical proxies in regulatory frameworks, although they do not directly represent the bioavailable fraction(*22*). As a result, the

modeling approach used here should be interpreted as a practical approximation rather than a direct representation of true bioavailability.

The model simulates annual changes in PFOS and PFOA concentrations in soil based on evolving soil pH, organic carbon content, and predicted sorption behavior. Partitioning between soil and water was governed by predicted K_{oc}, which were estimated as a function of pH using a site-calibrated regression described above. These K_{oc} values were then used to compute distribution coefficients (K_d) and dissolved PFOS concentrations, assuming equilibrium sorption.

PFOS and PFOA removal by plants was modeled separately for hemp and red fescue. Red fescue removal was applied as a fixed percentage drawn with variability from published experimental values(*21*). Each year, the remaining soil PFOS concentration was calculated by applying the combined removal of both plant species, adjusted to ensure a minimum 0.5% annual reduction to avoid unrealistic model outcomes at very high K_{oc} values.

Simulations were run for 20 years under a Monte Carlo framework to capture uncertainty in key parameters including K_{oc} variability, annual pH change, and red fescue uptake efficiency. A triangular distribution was used to simulate growing-season length to capture interannual variability in climate and crop growth duration. Plant uptake efficiency was scaled by the ratio of the modeled season length to the reference study's duration of 90 days (*15*). This was done so that empirical data used to predict removal rates were not limited to a 90 day growing cycle:

$$S_{hemp} = \frac{G_{\mathrm{S}}}{G_{\mathrm{ref}}} \quad (6)$$

$$R_{hemp,t} = f(K_{oc,t}) \cdot S_{hemp} \quad (7)$$

$$C_{soil,t} = C_{soil,t-1} \times (1 - R_{hemp}) \times (1 - R_{fescue}) \quad (8)$$

For the Monte Carlo analysis, 1,000 simulations were conducted by sampling from normal distributions for each uncertain parameter presented in Table S2. The model tracked PFOS and PFOA soil concentrations across all simulations, and outputs included the mean, 5th, and 95th percentile concentrations over time. A secondary scenario was also run in which K_{oc} was held constant (no pH effect) to isolate the impact of soil chemistry evolution.

1.8 Biochar Amendment and Sorption Coefficients

To evaluate the impact of biochar amendment on PFAS sorption, we modeled K_d dynamics over a 15-year period. The total soil mass per hectare was estimated as 3,900 tons, assuming a soil depth of 30 cm and a bulk density of 1,300 kg/m³ (*23*). To simulate the variability of PFAS sorption in amended soils, the model stochastically estimates the K_d using a combination of two components: (1) a probabilistic biochar-water coefficient (K_f) that is compound-specific, and (2) an adjustment factor based on the mass and effectiveness of biochar present in the soil.

The biochar sorption potential is represented by K_f, which is assumed to follow a normal distribution based on the range of values reported in the literature (*24–26*):

$$logK_f \sim \mathcal{N}\left(\log_{10}(\overline{K_f}), \sigma^2_{\log K_f}\right) \quad (9)$$

The estimated K_d for each iteration is then calculated by adjusting the stochastic K_f value with a biochar efficiency factor, which incorporates both the mass fraction of biochar in the soil and an efficiency correction designed to account for competitive sorption at lower biochar addition ranges (i.e., <1% biochar: soil). Table S3 summarizes the parameters used for these calculations. This comes from the literature on the effectiveness of biochar to limit plant uptake in amended soils (*25*, *27*, *28*). The effect typically seen is that biochar has a limited to no effect on the plant

uptake until a threshold is reached, likely due to competitive sorption within the soil/ porewater. Table S4 summarizes the results of existing studies exploring applied sorbents impact on plant uptake and is the basic for this model. To account for this lag in plant uptake potential the biochar's effectiveness on limiting plant uptake was corrected until 1% biochar by mass of reached, a value shown to limit uptake of PFOS (*28*).

$$\text{biochar percent}_t = \left(\frac{\sum_{i=1}^{t} \text{Mass}_{\text{biochar},i}}{\text{Mass}_{\text{soil}}}\right) \times 100 \qquad (10)$$

$$eff_b = \begin{cases} 0.5 \cdot \text{biochar\%}, & \text{if biochar\%} < 1 \\ 0.5 + (\text{biochar\%} - 1), & \text{if biochar\%} \geq 1 \end{cases} \qquad (11)$$

The $\text{eff}_{\text{biochar}}$ coefficient then is used to determine the K_d at the site with the new addition of biochar.

$$K_d = 10^{\log K_f} \times \left(\frac{\text{eff}_{\text{biochar}}}{100}\right) \qquad (12)$$

The new Kd is then added to the soil Kd at year 0 or with no biochar addition which is 10 L/Kg for PFOA and 100 L/Kg for PFOS based on literature values(*19*). To avoid unrealistically high sorption values resulting from the tail of the log-normal distribution, the model imposes an upper cap on K_d denoted as K_{dmax}.

As established before in section 1.6 the removal of PFAS via plant uptake was assumed to be inversely related to its sorption (i.e., compounds with lower sorption K_d remain more available in the soil solution and are more likely to be taken up by plants).To capture this relationship, we

applied an exponential decay function (Equation 13) that links the plant removal fraction to the effective K_d for each compound–biochar–soil combination. Where b is determined by solving for the rate constant which best fits the Kd to removal efficiency, as described in Section 1.7 This is the same Equation 5. used in Section 1.7 to model plant uptake for phytoremediation; however, instead of decreasing K_{oc} to enhance uptake, in this context, K_d is increased through the addition of biochar to reduce plant uptake. To ensure model predictions remained realistic, we incorporated a scaling parameter (min_eff) into the exponential uptake equation. This value represents the minimum observed plant removal efficiency at the highest measured K_d and reflects the fact that PFAS uptake by plants rarely reaches zero. By anchoring the exponential decay curve with min_eff, the model captures both the empirical lower bound of uptake and the diminishing bioavailability of PFAS as sorption increases (Fig S6).

$$\text{Removal}_t = \text{min\_eff} \times \exp\left(b \times \left(K_d - K_{d,\max}\right)\right) \quad (13)$$

1.9 Risk based thresholds for plant concentrations

To contextualize the significance of limiting PFOS uptake into plant biomass through biochar amendment, we compared the modeled PFOS concentrations in hemp to risk-based thresholds developed for the agronomic exposure pathway of soil to milk through grazing on impacted land.(*29*)

The most stringent, available screening level was the Maine CDC's PFOS soil screening level (SSL) for dairy farms, which is based on preventing exceedance of a milk PFOS action level of 210 ng/L (*29*). While SSLs are reported as soil concentrations, the derivation methods include published soil-to-plant transfer factors (TF_{plant}) and mass loading factors (MLF), which were

used to back-calculate a plant tissue threshold corresponding to this milk protection goal. These parameters are summarized in Table S5. The equation below was applied to convert the soil-based SSL into a corresponding PFOS concentration in plant tissue (dry weight)

$$C_{plant} = C_{soil} * \left(TF_{plant} + MLF\right) \quad (14)$$

1.10 Enhanced weathering CDR estimates

CDR from ERW was estimated using the reactive transport model SCEPTER(*30–32*). Kanzaki et al. (2025) conducted ensembles of ERW simulation on CONUS croplands (defined as the area where >10% land use is for cropland) at 1°x1° resolution. Their experiments were repeated here but extended to all CONUS areas. In a brief summary, ERW experiment procedure was as follows:

(1) models were initialized to reproduce observed soil pH, base saturation, soil organic carbon contents, and soil pCO_2.
(2) Two ensembles of ERW experiments were conducted for 100 model years, by adding either CaO or basalt, to the initialized models so that at the end of each year, target soil pH = 7 is satisfied.

We utilized 1°x1° grid products used by Kanzaki et al. (2025) as input to the model, including temperature, soil moisture, runoff/infiltration, cation exchange capacity, nitrification rate, soil erosion, soil porosity, net primary production (NPP) (see their Table 1), excluding those used as observation targets for the model to reproduce (soil pH, base saturation, soil organic carbon contents, and soil pCO_2 which is calculated with NPP and temperature). Model setup is simplified as described in Kanzaki et al. (2024, 2025) where simulated solid phases are limited to a bulk soil phase plus a soil organic matter both of which are assumed to have the same cation

exchange properties. Aqueous species tracked in the model include Ca, Mg, Na, K, Si and NO_3 and gaseous CO_2 is tracked as well. Model's boundaries are specified with fixed compositions at the top for aqueous and gaseous phases, while at the bottom for solid phase where gaseous and aqueous diffusions are not allowed. Four model parameters are tuned during the initialization process [(1) above]. They include a parameter for cation exchange between Na and H, Ca concentration at the top boundary, organic matter input to the model and turnover time for organic matter. Cation exchanges among cations (e.g., Ca-H, Mg-H) are all scaled with the Na-H exchange so tuning of the Na-H exchange parameter, along with Ca concentration at the top boundary, enabled the model to reproduce observed soil pH and base saturation. Tuned Ca concentrations can be regarded as representing cation inputs to the model from the historical ag liming plus background weathering. See Supplementary material of Kanzaki et al. (2025) for more details on the tuned parameters.

In the model, soil CO_2 was tracked which enabled direct estimation of CDR either through reduction of CO_2 emission to the atmosphere or increase of DIC through porewater advection, which converge within a couple of porewater residence time. We take the former method (CDR as reduction of soil CO_2 emission) for our CDR calculation. This choice does not affect our CDR calculation by ERW.

1.11 Transport of ERW feedstock estimates

To model transport for EW material, stochastic ranges were established using existing literature to simulate three scenarios—semi-truck transport, semi-truck and barge transport, and semi-truck and rail transport. This was done as a constraint on deployment feasibility rather than to directly estimate transport-related emission losses which were assumed to be 10% taken from an LCA on

EW deployment in the U.S. (*33*). To calculate the amount of rock material available for EW application M, the maximum load of a semi-truck L_T was multiplied by the inefficiency due to the grinding process i_g. From equation 15, the available ion content was calculated by multiplying M by the inefficiency resulting from losses of ions to the ocean, representing the initial weathering potential of the parent material.

$$M = L_T \times \left(1 - i_g\right) \qquad (15)$$

$$C_{available} = M \times (1 - i_o) \qquad (16)$$

Equation 15 calculates the potential CDR of EW application of rock material transported. To do this, the result of Equation 16 is multiplied by C_c, the fraction of the parent material weatherable for CDR, which has a stochastic range of 0.20 to 0.30(*33*, *34*). This is assumed to represent the tons of CO_2 removed upon basalt application at the site.

$$CDR_{site} = C_{available} \times C_c \qquad (17)$$

To find the emissions associated with transport of ERW material, scenarios were simulated assuming a fully loaded semi-truck transporting material to the application site. Fuel efficiency was established in two stochastic ranges for a fully loaded truck F_f, and an empty truck on return trips F_e, with values of 9.66–12.87 km/gal and 10.46–12.87 km/gal, respectively. respectively(*35*). The CO_2 emissions for diesel fuel E_d were established from the literature at 10.19 kg CO_2/gal . Equations 18 and 19 show the emissions for a full (E_{km}) and empty ($E_{km\ empty}$) semi truck. A 25% discount was applied to emissions for the return, under the assumption that industrial transport operates as a network, making some trips one-way.

$$E_{km} = \frac{E_d}{F_f} \qquad (18)$$

$$E_{km\ empty} = 0.75 \times \frac{E_d}{F_e} \qquad (19)$$

Total emissions for truck transport were then calculated using the emissions for each trip multiplied by the trip distance d_t. Trip distances were kept in the stochastic range of 10 to 1000 km.

$$E_{total\ truck} = (E_{km} \times d_t) + (E_{km\ empty} \times d_t) \qquad (20)$$

To establish a percentage inefficiency due to transport e_r, the total emissions from truck transport were divided by CDR potential without any transport emissions ($CDR_{potential}$).

$$e_r = \frac{E_{total\ truck}}{CDR_{potential}} \qquad (21)$$

An index of acceptable scenarios from the Monte Carlo simulation were created, limiting the sum of the inefficiencies e_r,i_o, and i_g to equal to the target inefficiency i_t, with a ±1% tolerance range. Target inefficiencies of $i_t = 0.25$ and $i_t = 0.30$ were simulated to produce two maximum distances.

$$-0.01 \leq (e_r + i_o + i_g) - i_t \leq 0.01 \qquad (22)$$

For barge transport, emissions were calculated utilizing a fuel efficiency metric F_B of 1086.3 km/gal/ton (*36*). To calculate efficiency per distance $F_{B\ cargo}$, the unit fuel efficiency was multiplied by the sum of the load L_T and the weight of the barge container $w_B$in Equation 8. The same load L_T is utilized in calculating semi-truck transport, as it is the limiting factor in cargo capacity for one trip to and from barge transport.

$$F_{B\ cargo} = F_B \times (L_T + w_B) \quad (23)$$

To calculate the total emissions of barge transport $E_{total\ barge}$, the rate of emissions per kilometer was first calculated in Equation 24 by dividing the emissions from one gallon of diesel fuel E_d by the fuel efficiency of material transport $F_{B\ cargo}$. Multiplying this quotient by the distance transported by barge d_b in Equation 23 results in the total emissions of barge transport $E_{total\ barge}$. d_b was assigned the stochastic range of 200-3000 km.

$$E_{km\ barge} = \frac{E_d}{F_{B\ cargo}} \quad (24)$$

$$E_{total\ barge} = \left(E_{km\ barge} \times d_b\right) \qquad (25)$$

Semi-truck emissions were calculated using the same method as detailed in the previous section, with an alteration of its stochastic range d_T to [10 , 50]. The emissions ratio $e_{r\ barge}$ was calculated by dividing the sum of the truck emissions to barge transport and total emissions resulting from barge transport by potential CDR.

$$e_{r\ barge} = \frac{\left(E_{total\ truck} + E_{total\ barge}\right)}{CDR_{potential}} \qquad (26)$$

An index of acceptable scenarios from the Monte Carlo simulation were created, limiting the sum of the inefficiencies $e_{r\ barge}$,i_o, and i_g to equal to the target inefficiency i_t, with a ±1% tolerance range (Equation 20). Target inefficiencies of $i_t = 0.25$ and $i_t = 0.30$ were simulated to produce two maximum distances.

For rail transport, emissions rates E_R were calculated from an established emissions range of 0.0002-0.00004 tCO2/(t*km) (*37*). To calculate emissions per distance $E_{km\ rail}$, E_R was multiplied by the sum of the load L_T and the weight of the barge container w_R. The same load L_T

is utilized in calculating semi-truck transport, as it is also the limiting factor in cargo capacity for one trip to and from rail transport.

$$E_{km\,rail} = E_R \times (L_T + w_R) \quad (27)$$

To calculate the total emissions of rail transport $E_{total\,rail}$, $E_{km\,rail}$ was multiplied by the distance transported by rail d_r in Equation 13. d_r was assigned the stochastic range of 100 to 1000 km.

$$E_{total\,rail} = (E_{km\,rail} \times d_r) \qquad (28)$$

Semi-truck emissions were calculated using the same method as detailed in the previous section, with an alteration of its stochastic range d_T to [10 , 50]. The emissions ratio $e_{r\,rail}$ was calculated by dividing the sum of the truck emissions to rail transport and total emissions resulting from rail transport by perfectly efficient CDR.

$$e_{r\,rail} = \frac{(E_{total\,truck} + E_{total\,rail})}{CDR_{perfect}} \qquad (29)$$

An index of acceptable scenarios from the Monte Carlo simulation were created, limiting the sum of the inefficiencies $e_{r\,rail}$,i_o, and i_g to equal to the target inefficiency i_t, with a ±1% tolerance range. Target inefficiencies of $i_t = 0.25$ and $i_t = 0.30$ were simulated to produce two maximum distances.

1.12 Biochar CDR Estimation

We estimated the potential net CDR from biochar production applied across impacted agricultural land throughout the contiguous United States. This analysis incorporated spatial variability in biomass productivity and amount of PFAS impacted land to produce biomass

yields. Impacted land area and its spatial location was determined as previously described above in section S1.2. In short the median amount of impacted land determined at 1.22 million hectares was broken into 1,000 hectare grid cells and distributed into states based on biosolid application data, then distributed within each state based on population densities. For each 1,000-hectare grid cell, we calculated the net CDR using Monte Carlo simulation of biomass conversion and emissions factors.

To reflect variation in growing season and biomass productivity, biomass yield (Y_b) was scaled linearly with latitude for each coordinate in the dataset. Latitude was normalized across the national domain, and yields were interpolated between a minimum and maximum value:

$$Y_{\mathrm{b}} = Y_{\max} - \left(\frac{\mathrm{lat}_{\max} - \mathrm{lat}_{\min}}{\mathrm{lat} - \mathrm{lat}_{\min}}\right) \cdot (Y_{\max} - Y_{\min}) \quad (30)$$

The yield for that specific grid cell was then used to determine the mass of biochar produced using the area of the total grid cell (1,000 ha) and the Biochar conversion efficiency (η) of the pyrolizer:

$$B_{\mathrm{char}} = Y_b \cdot A \cdot \eta \quad (31)$$

The CDR of the biochar at each cell was then determined by converting biochar to its equivalent mass of carbon and the stoichiometric ratio of C to CO_2 equal to 3.67:

$$\mathrm{CDR}_b = \mathrm{B}_{char} \cdot \mathrm{C}_f \cdot 3.67 \quad (32)$$

Losses were also accounted for from pyrolysis and transport of biomass and biochar. The median transport losses were ~1.5 %. Transport emissions were estimated by calculating the number of

truck trips required to move biomass, multiplied by round-trip distance and fuel efficiency. Emissions were then derived from the diesel emission factor, where C_{truck} is truck capacity (t/load), d is the round-trip transport distance (km), F_f is the vehicle fuel efficiency (L km^{-1}), and ϵ_{diesel} is the diesel emission factor (kg CO_2 L^{-1}).

$$\text{Trips} = \frac{B}{C_{\text{truck}}} \quad (33)$$

$$E_{\text{transport}} = \text{Trips} \cdot d \cdot F_{\text{f}} \cdot \epsilon_{\text{diesel}} \quad (34)$$

Pyrolysis emissions were expressed as the emissions per ton of biomass processed were ϵ_{pyro} is the pyrolysis emission rate (kg CO_2 t^{-1}). Emission factors were derived from literature values, with reported ranges reflecting variation in process design, energy inputs, and operational conditions. These values are based on LCA studies that differ in system boundaries. For example, some include only direct CO_2 emissions from reactor operation, while others incorporate additional sources such as capital goods, pre-heating, and electricity use, and may report CO_2-equivalent (CO_2e) emissions including non-CO_2 greenhouse gases.

Given this variability, emission factors from different studies were used to define a reasonable range rather than a single point estimate. This analysis is not intended to represent a strict, ISO-compliant LCA, and therefore integrates values across studies with differing system boundaries. As a result, the reported range should be interpreted as an approximate estimate and could be

further refined in future work through process-specific LCAs with a clearly defined and consistent system boundary.

$$E_{\text{pyrolysis}} = \epsilon_{\text{pyro}} \cdot B \qquad (35)$$

These two sources of loss were then subtracted from the total CDR calculated from the biochar production to result in a net CDR.

$$\text{CDR}_{net} = \text{CDR}_{biochar} - \text{e}_{trucking} - \text{e}_{pyrolysis} \qquad (36)$$

We acknowledge that long term/ permanent storage would require an in-depth assessment of decay rates across the U.S. and with production temperatures so a 18-37% loss was then assumed for all CDR following application (*38*). Emissions associated with pyrolysis were assumed to arise from fossil energy inputs required for system operation (e.g., diesel use, auxiliary heating). Biogenic CO_2 released during thermal decomposition of biomass was not counted as a net emission, as it represents recently fixed atmospheric carbon. Net CDR was therefore calculated as the CO_2-equivalent of carbon retained in biochar minus fossil emissions associated with transport and operation. The resulting CDR was then assumed to be long term storage. All parameters for these calculations are presented in Table S6 below.

1.13 Traditional remediation emission estimates and costs

To model transport for PFAS contaminated soils to traditional remediation sites, stochastic ranges were established using existing literature to simulate two scenarios: semi-truck transport and semi-truck and rail transport. Models simulated transport to a hazardous waste landfill, as well as a rotary kiln, to show alternative scenarios and the associated emissions of carbon dioxide.

Semi-truck transport was calculated using the same method as calculations done for the transport of biomass material in an earlier section. The same load size, fuel efficiencies, and emissions due to the combustion of diesel fuel were used. Semi-trucks were assumed to be fully loaded, with a large discount on backhauls assuming a short reposition distance d_d for the next job. To calculate the number of farms impacted by PFAS contamination across the United States, the 1.2 million ha of impacted land i calculated previously was divided by the average farm size a_f:

$$f_i = \frac{i}{a_f} \quad (37)$$

The weight of soil per ha was then calculated, multiplying average soil density by the depth of soil and the conversion factor. To then find the amount of contaminated soil per farm we multiplied the average farm size by the unit weight of contaminated soil:

$$w_s = s_d \times \rho_s \times 1000 \frac{m^2}{ha} \quad (38)$$

$$s_f = w_s \times a_f \quad (39)$$

After finding the total amount of soil removal on the individual farm scale, the number of trips required for transport of the soil to an external site was calculated by dividing the amount of soil per farm by the maximum load size of a semi-truck and rounded up to the nearest whole number.

$$t_f = \frac{s_f}{L_T} \qquad (40)$$

To simulate the transport of contaminated soil to a hazardous waste landfill, distances d_l between were simulated stochastically. The emissions for each trip were calculated with average emissions for a full semi-truck being multiplied by the trip distance, as in section 1.11 trucking emissions. This product was then multiplied by the trips required per farm to get an estimate of total emissions per farm due to transport to hazardous landfill. If the distance exceeded the maximum of 120 km it was assumed a more efficient rail pathway could have been selected and rail emissions were estimated. For a unit estimate of emissions, the total truck emissions per farm was divided by the average farm size to produce emissions per hectare:

$$E_{trip\ truck\ l} = E_{km\ truck} \times d_l \qquad (41)$$

$$E_{truck\ l} = E_{trip} \times t_f \qquad (42)$$

$$E_{ha,truck,l} = \frac{E_{total\ truck\ l}}{a_f} \qquad (43)$$

$$E_{rail\ l} = E_R \times t_r \qquad (44)$$

The second scenario for contaminated soil removal involves transport of the material to a rotary kiln for thermal degradation of PFAS. Emissions associated with this process included both transport of the material to the facility as well as the emissions due to operation of the kiln. Calculations of emissions due to operation of the kiln were calculated using energy consumption

and associated emissions ranges. To calculate the number of operational hours required to process a farm's contaminated soil, the total soil per farm was divided by the kiln's hourly capacity:

$$t_k = \frac{s_f}{c_k} \qquad (45)$$

The energy consumption and associated emissions of running the kiln were then calculated by using a BTU usage (MMBtu/hr) for processing contaminated soils, assumed based on literature reports see Table S7. To calculate BTUs required per farm, the amount of time to process a farm's contaminated soils was multiplied by a kiln's hourly BTU consumption. The associated emissions were calculated using a range of emissions from differing fuel sources multiplied by the BTU usage per farm.

$$BTU_{farm} = u_{BTU} \times t_k \qquad (46)$$

$$E_k = E_{BTU} \times BTU_{farm} \qquad (47)$$

Transport was calculated in the same manner as for the landfill scenario, but with a different trip distance range. Additionally, the kiln was assumed to oxidize all soil organic matter to CO_2, adding to the total emission burden. This was calculated by multiplying the total soil mass by the fraction of organic matter f_{OM}, the fraction of carbon within the organic matter $f_{C|OM}$, and the stoichiometric conversion factor from carbon to CO_2 (≈3.67 by mass).

$$E_{OM} = s_f * f_{OM} * f_{C|OM} * 3.6 \qquad (48)$$

The total emissions associated with truck transport for soil remediation via rotary kiln was first calculated at a farm level, taking the sum of the emissions due to kiln operation and material

transport to the facility. A unit estimate was then taken by dividing the calculated value by the average farm size. The results of these emission estimates are shown in Fig S7.

$$E_{total\ truck\ k} = E_k + E_{truck\ k} \qquad (49)$$

$$E_{ha,truck,k} = \frac{E_{total\ truck\ k}}{a_f} \qquad (50)$$

Cost estimates were based on prior work that established unit cost benchmarks for the remediation and disposal of PFAS-contaminated soils under U.S. Superfund site management(*39*). The previously estimated total soil mass across 1.2 million ha of impacted land was multiplied by the per-ton cost estimates from that report, resulting in approximately $1.3 trillion for hazardous-waste landfill disposal and $2.4 trillion for thermal treatment (Fig S8).

1.14 Cost of Biochar Production

Biochar production cost analyses were simulated under two conditions. Large pyrolysis facilities operated under the assumption of requiring material transport—transporting biomass and returning biochar—by semi-truck. Mobile pyrolysis rigs operated without transport requirements, but included labor, fuel and start-up costs. To capture technical and economic differences between scenarios, the unit cost per ton of CDR was calculated and depicted on a histogram in Fig S8. All values or ranges for parameters are in Table S8.

For large pyrolysis facilities, the biomass consumption rate $B_L$and hourly operational cost $O_L$were defined as stochastic input parameters. Hourly biochar production $c_{h,L}$was determined by multiplying the biomass input rate by the biochar yield factor b_{bc}. The resulting hourly production was scaled by the total operating hours of the facility $T_L$to obtain daily biochar output

$c_{d,L}$. The corresponding daily biomass throughput $B_{d,L}$ was similarly computed as the product of the biomass input rate and operating time.

$$c_{h,L} = B_L \times b_{bc} \qquad (51)$$

$$c_{d,L} = c_{h,L} \times T_L \qquad (52)$$

$$B_{d,L} = B_L \times T_L \qquad (53)$$

The carbon content of the produced biochar $C_{d,L}$ was calculated by multiplying the daily biochar production $c_{d,L}$ by the biochar carbon fraction C_{BC}. The total potential carbon dioxide removal $CDR_{d,L}$ was then determined by converting the stored carbon to its equivalent mass of CO_2 using the stoichiometric conversion constant c_{cc}.

$$C_{d,L} = c_{d,L} \times C_{BC} \qquad (54)$$

$$CDR_{d,L} = C_{d,L} \times c_{cc} \quad (55)$$

Economic analyses of biomass markets provided stochastic estimates of the unit biomass price P_B, which was used to calculate the total daily feedstock cost for the facility. The daily biomass cost $P_{B,d,L}$ was determined by multiplying the unit biomass price by the total biomass processed $B_{d,L}$, assuming continuous operation at full capacity.

$$P_{B,d,L} = P_B \times B_{d,L} \qquad (56)$$

Because large stationary pyrolysis systems require external transport, material delivery and product return were incorporated into the model. The number of daily biomass deliveries $t_{d,L}$ was calculated by dividing the total daily biomass processed $B_{d,L}$ by the truck load capacity L_T.

Return trips for biochar transport were determined using the same approach, substituting daily biochar production $c_{d,L}$ for biomass throughput.

$$t_{d,L} = \frac{B_{d,L}}{L_T} \qquad (57)$$

$$t_{return\ d,L} = \frac{c_{d,L}}{L_T} \qquad (58)$$

Emissions associated with transport were estimated following the same methodology used in the transport economics framework. The emission rate per kilometer E_{km} was calculated by dividing the diesel emission factor E_d by the average fuel efficiency of a fully loaded truck F_f. Emissions for each trip E_{trip} were then obtained by multiplying the per-kilometer emission rate by the transport distance d_t. The total transport emissions E_{total} for the large pyrolysis facility were determined by summing the emissions from both biomass delivery trips $t_{d,L}$ and biochar return trips $t_{return,d,L}$.

$$E_{trip} = E_{km} \times d_t \qquad (59)$$

$$E_{total} = (E_{trip} \times t_{d,L}) + (E_{trip} \times t_{return\ d,L}) \qquad (60)$$

Transportation costs were estimated using a per-distance operating cost for semi-truck transport P_{km}, derived from literature sources. The total cost per trip P_{drive} was calculated by multiplying P_{km} by the transport distance d_t. Daily transport cost $P_{transport}$ was then determined by multiplying P_{drive} by the number of biomass delivery trips $t_{d,L}$ and biochar return trips $t_{return,d,L}$, and summing the two components to represent the total transport expense for a fully operational facility.

$$P_{transport} = \left(P_{drive} \times t_{d,L}\right) + \left(P_{drive} \times t_{return\ d,L}\right) \qquad (61)$$

Total system cost calculations incorporated both capital cost, and operational and material expenses. In large pyrolysis systems, co-products (e.g., syngas, bio-oil) can be combusted to supply process heat and power, allowing operations to be partially or fully energy self-sufficient. Here, we assume co-products are used primarily for internal energy recovery rather than external sale. We used capital cost data from a previous study(*40*) for a woody biochar pyrolysis plant with a capacity of 2,000 dry t feedstock/day, 330 days/year operation, 10% discount rate, and 20 years lifetime: the capital cost is $70/t biochar.

The daily operational cost $O_{d,L}$ was determined by multiplying the facility's operating time T_L by its stochastic hourly operational cost O_L. The overall daily cost $P_{total,L}$ was then computed as the sum of transport costs $P_{transport}$, operational costs $O_{d,L}$, and biomass feedstock costs $P_{B,d,L}$, representing the total expenditure required for continuous operation of the large pyrolysis facility

$$P_{total,L} = P_{transport} + O_{d,L} + P_{B,d,L} \quad (62)$$

The unit cost of carbon dioxide removal $P_{CDR,L}$ for large pyrolysis facilities was calculated by dividing the total daily system cost $P_{total,L}$ by the net amount of carbon dioxide removed $CDR_{net,L}$. This metric represents the cost per ton of CO_2 effectively sequestered after accounting for all emissions and operational expenses associated with the process.

$$P_{CDR,L} = \frac{P_{total,L}}{CDR_{net,L}} \qquad (63)$$

For mobile pyrolysis units, the biomass consumption rate B_M and hourly operational cost O_M were defined as stochastic input parameters to reflect variability in small-scale system performance. Calculations followed the same methodology as those for the large stationary facility. Hourly biochar production $c_{h,M}$, daily biochar production $c_{d,M}$, and total daily biomass processed were

computed using the corresponding relationships among biomass input rate, conversion efficiency, and operating hours T_M. The range of operational hours for mobile units was defined to capture typical variability observed across field-scale deployments.

$$c_{h,M} = B_M \times b_{bc} \qquad (64)$$

$$c_{d,M} = c_{h,M} \times T_M \qquad (65)$$

$$B_{d,M} = B_M \times T_M \qquad (66)$$

The carbon content of the biochar, overall carbon dioxide removal potential, and daily biomass cost for mobile units were calculated using the same relationships established for the large facility. Specifically, the carbon content was determined from the daily biochar production and carbon fraction, the CDR potential was obtained by converting stored carbon to its CO_2 equivalent, and the daily biomass cost was calculated as the product of unit biomass price $P_B$and total biomass processed $B_{d,M}$.

$$C_{d,M} = c_{d,M} \times C_{BC} \qquad (67)$$

$$CDR_{d,M} = C_{d,M} \times c_{cc} \qquad (68)$$

$$P_{B,d,M} = P_B \times B_{d,M} \qquad (69)$$

Mobile pyrolysis units offer the advantage of on-site operation, eliminating the need for transport of biomass or biochar and thereby reducing associated emissions and costs. Consequently, their economic calculations differ from those of large stationary systems. Fuel use was incorporated as a primary operational expense, with average hourly fuel consumption $F_M$used to estimate total daily fuel use $F_{d,M}$by multiplying $F_M$by the total operational hours T_M. The corresponding daily

fuel cost $P_{F,d,M}$ was then calculated by multiplying the total fuel consumed by the unit price of diesel fuel P_{fuel}.

$$F_{d,M} = F_M \times T_M \qquad (70)$$

$$P_{F,d,M} = F_{d,M} \times P_{fuel} \qquad (71)$$

Purchasing a mobile pyrolysis unit represents a significant upfront capital investment P_M, which includes equipment cost, installation, and operator training. To incorporate this expense into the daily cost framework, the total capital cost was first annualized using a capital recovery factor and then converted to a daily equivalent investment cost by dividing the annualized capital cost by the number of operating days per year for the mobile system. Levelized capital cost was calculated as the product of the capital cost and the capital recovery factor(*41*):

$$\text{CRF} = \frac{i(1+i)^n}{(1+i)^n-1} \qquad (72)$$

where i is the discount rate and n is the project lifetime. A discount rate of 10% and amortization period of 30 years were assumed based on a previous biomass gasification LCA(*41*). The resulting annualized capital cost was then converted to a daily equivalent capital cost by dividing by the assumed number of annual operating days for the mobile system:

$$P_{M,\text{daily}} = \frac{P_M \times \text{CRF}}{D_M} \qquad (73)$$

Total daily system costs for the mobile pyrolysis unit $P_{total,M}$ were calculated as the sum of daily equivalent capital cost $P_{Minitial}$, operational costs $O_{d,M}$, biomass feedstock costs $P_{B,d,M}$, and daily fuel costs $P_{F,d,M}$. Emissions from the mobile system $E_{d,M}$ were estimated using fuel efficiency data, with the diesel emission factor E_d divided by the fuel efficiency F_M and multiplied by the total operational hours T_M to determine daily CO_2 emissions associated with system operation.

$$P_{total,M} = P_{M,daily} + O_{d,M} + P_{B,d,M} + P_{F,d,M} \quad (74)$$

$$E_{d,M} = \frac{E_d}{F_M} \times T_M \quad (75)$$

Net carbon dioxide removal $CDR_{net,M}$ and the corresponding cost per ton of removal $P_{CDR,M}$ for mobile pyrolysis units were calculated using the same methodology applied to the large facility. Net CDR was determined by subtracting operational emissions $E_{d,M}$ from the gross CDR potential, and the unit cost of removal was obtained by dividing the total daily system cost $P_{total,M}$ by the net amount of CO_2 removed.

$$CDR_{net,M} = CDR_{d,M} - E_{d,M} \quad (76)$$

$$P_{CDR,M} = \frac{P_{total,M}}{CDR_{net,M}} \quad (77)$$

In addition to biomass feedstock costs, a preprocessing cost was applied on a per-ton biomass basis to account for field-collected material requiring size reduction prior to pyrolysis. Because biomass was assumed to be field-dried prior to transport, no additional drying cost was included. Instead, for large scale facilities only preprocessing costs represent grinding, handling, and conditioning of biomass prior to reactor feeding. No preprocessing was accounted for small scale

units. Additionally, a fraction of harvested biomass was assumed to require temporary storage prior to processing. Storage costs were applied on a per-ton basis and represent handling, staging, and short-term buffering of biomass supply due to seasonality. A small dry matter loss (1–3%) was applied to account for degradation during storage. Residual solids generation during pyrolysis was represented as a fraction (1–2%) of the processed biomass mass. A per-ton handling cost was applied to this residual fraction to account for disposal or management of ash and non-carbonized material streams. These costs were included as part of the total system cost but were not assumed to contribute to carbon storage.

These losses and costs were added to the cost of processing each ton of biomass and summed up to get the total cost per ton of biochar produced. Results of the cost per ton of CDR for each method are displayed in a histogram in Fig S9.

1.15 Remediation cost estimates

For the net annual cost of remediation (NC), a stochastic framework was developed to account for both costs and revenues associated with the proposed approach. The model incorporated: (i) annual operational costs for cultivating hemp and fescue/hay, (ii) annual payments to farmers for participation and labor, (iii) deployment costs for biochar and enhanced rock weathering (ERW), and (iv) revenues from the sale of CDR credits generated from biochar and ERW. Deployment costs and CDR credit revenues for each technology were scaled according to the CDR rate, reflecting their correlation (e.g., reduced biomass production lowers transport and operational costs but also decreases biochar output and total CDR achieved). This net annual cost was then calculated per iteration (N = 100,000) and per year (t). in the equation below:

$$NC_{i,t} = C_{\text{hay},t} + C_{\text{hemp},t} + I_{\text{farmer},t} + P_{\text{biochar},i} \cdot Q_{\text{biochar},t} + P_{\text{ERW},i} \cdot Q_{\text{ERW},t} - S_{\text{biochar},t} \cdot Q_{\text{biochar},t} - S_{\text{ERW},t} \cdot Q_{\text{ERW},t} \quad (78)$$

The total remediation cost was calculated by summing the net annual remediation costs over the duration of each scenario (T), where T was drawn from a truncated normal distribution (μ = 22.5, σ = 4.5) bounded between 10 and 60 years to reflect realistic deployment limits (parameters shown in Table S9).

$$TC_i = \sum_{t=1}^{T_i} NC_{i,t} \quad (79)$$

1.16 Breakeven cost scenarios

As the price of CDR credits from biochar and ERW sales directly influences the net annual cost of remediation, this parameter was explored to show have prices of CDR influence the remediation costs. In the model, assumed CDR prices were based on current market sales; however, these values do not account for potential overpayments an organization might accept in exchange for the co-benefits of PFAS remediation and supporting small farmers. To explore this effect, the total average sale price of CDR credits was varied and plotted against the total remediation cost to assess how market price fluctuations influence the financial burden of remediation. The first step in this process was to calculate the average CDR price using the equation below:

$$P_{\text{combined},i} = \frac{S_{\text{biochar},i} \cdot Q_{\text{biochar},i} + S_{\text{ERW},i} \cdot Q_{\text{ERW},i}}{Q_{\text{biochar},i} + Q_{\text{ERW},i}} \quad (80)$$

Once a combined CDR price was calculated, it was incorporated into the above equation to estimate the annual net costs across a range of scenarios, from no carbon credit revenue (i.e.,

zero CDR sales) to an upper bound of 275 USD t^{-1}, at which point average remediation costs approached zero. This analysis was performed stochastically, with 1,000 price points uniformly sampled between 0 and 275 USD t^{-1}. For each price point, 400 simulations were conducted, and the mean net cost was recorded. The standard deviation across simulations was used to derive the 10th and 90th percentile ranges for each price level. The results of the are shown in Fig S10.

1.17 Pyrolysis Emissions and Infrastructure Requirements for Biomass Processing

To evaluate whether the proposed remediation strategy is constrained by pyrolysis infrastructure capacity, we estimated the total biomass requiring annual processing and compared it to the throughput of representative pyrolysis systems.

The total mass of biomass generated annually across PFAS-impacted agricultural land was calculated using the estimated impacted area (Section 1.2) and biomass yield assumptions (Table S6). With a median impacted land area of 1.22×10^6 hectares and biomass yields ranging from 10–18 metric tons ha^{-1} yr^{-1}, the total annual biomass requiring processing (B_{total}) is:

$$B_{\text{total}} = A_{\text{impacted}} \times Y_b \qquad (81)$$

resulting in a range of 12.2–30.5 million metric tons yr^{-1}, with a central estimate of approximately 21.35 million metric tons yr^{-1}.

To estimate the annual throughput of a single pyrolysis facility, we used the biomass consumption rates and operating conditions defined in Section 1.14. Facilities were assumed to operate at 95% uptime, corresponding to 346 days a year of operation. For large stationary facilities, the annual biomass processing capacity ($B_{facility}$) was calculated as:

$$B_{\text{facility}} = B_{\text{rate}} \times H_{\text{op}} \times D_{\text{op}} \quad (82)$$

where B_{rate} is the biomass feed rate (30–40 t hr^{-1}) and H_{op} is the daily operating time (8–24 hr day^{-1}). This results in an annual throughput range of approximately 8.3×10^4 to 3.3×10^5 metric tons yr^{-1} per facility, with a central estimate of ~1.9×10^5 metric tons yr^{-1}.

The number of required large facilities (N_{facility}) to process all biomass annually is then:

$$N_{\text{facility}} = \frac{B_{\text{total}}}{B_{\text{facility}}} \quad (83)$$

Using the ranges above, this yields approximately 35–360 large stationary facilities, with a mean estimate of 110 facilities required to process all biomass generated annually.

For mobile pyrolysis units, using biomass processing rates of 1–4 t hr^{-1} and operating times of 6–8 hr day^{-1}, the annual throughput per unit is estimated to range from approximately 2.1×10^3 to 1.1×10^4 metric tons yr^{-1}. Under these assumptions, processing the full biomass stream would require approximately 1,100–8,000 mobile units, with a mean estimate of ~3,500 units.

These estimates represent first-order infrastructure requirements under idealized operating conditions and do not explicitly account for several important factors that could influence system feasibility and cost. In particular, the analysis does not incorporate detailed financial structures, labor costs, or capital cost scaling as a function of facility capacity. Additionally, process-level considerations including reactor design, system integration, and the potential co-production of biochar and other value-added products are not explicitly resolved. A more comprehensive

techno-economic assessment incorporating discounted cash flow analysis would be required to fully evaluate system-level costs.

For infrastructure availably, these estimates do not explicitly account for spatial coordination, downtime beyond the assumed uptime fraction, feedstock logistics, or regional clustering of facilities, all of which could influence deployment efficiency and economics. As such, the results should be interpreted as order-of-magnitude estimates intended to assess infrastructure requirements rather than definitive projections of system performance or cost. However, they provide a useful benchmark for evaluating whether sufficient processing capacity exists to support large-scale deployment.

While these infrastructure requirements are substantial, they are not unrealistic given the pressures to expand biochar/ activated carbon or biofuels capacity in the context of environmental regulation-driven technology deployment. Recent and proposed drinking water standards for PFAS at ng L^{-1} levels(*42*) are expected to significantly increase demand for sorptive treatment media, particularly granular activated carbon (GAC), which already relies on large-scale, continuous thermochemical production systems(*43*). Achieving compliance at these low concentrations necessitates frequent media replacement, resulting in high material throughput demands across municipal and industrial water treatment systems(*44*). As regulatory pressure expands, both activated carbon and emerging alternatives such as biochar are expected to require significant expansion in production capacity. In this context, the number of facilities estimated here is comparable to the scale of infrastructure development already underway or anticipated for PFAS treatment in the water sector, suggesting that the required buildout, while large, is consistent with broader regulatory-driven industrial scaling.

PFAS concentrations commonly observed in agricultural systems (low µg/kg range) are relevant from a food-chain and human exposure perspective due to their bioaccumulation potential; however, the total fluorine inventory associated with these concentrations remains small in an absolute mass-balance sense. For example, assuming an intentionally conservative upper-bound case of 100 µg/kg PFOS in biomass, one metric ton of material would contain approximately 100 mg PFOS, corresponding to roughly 65 mg of fluorine. If all fluorine were unrealistically converted to HF, the maximum possible yield would be approximately 68 mg HF per metric ton of biomass. When diluted in the exhaust stream of a thermal treatment system, this would correspond to extremely low concentrations. In addition, HF is highly reactive and would be expected to associate rapidly with alkaline mineral phases present in biomass ash (e.g., Ca^{2+}), forming stable fluoride salts (e.g., CaF_2) that partition to solids or are captured in particulate control systems.

A similar upper-bound consideration applies to the potential formation of fluorinated gases such as tetrafluoromethane (CF_4), which has a high global warming potential ($GWP_{100} \approx 7{,}380$ (*45*)). Using the same 100 µg/kg PFOS scenario, complete conversion of all fluorine to CF_4 would produce approximately 75 mg CF_4 per metric ton of biomass, corresponding to roughly 0.5 kg CO_2-equivalent per ton. This is substantially smaller than typical greenhouse gas emissions associated with biomass transport or thermal treatment (on the order of 10–100 kg CO_2 per ton). These estimates represent a deliberately conservative upper bound, as they assume complete conversion of PFOS-derived fluorine into a single volatile product with no retention in char, ash, mineral phases, or pollution control systems. In practice, actual formation of HF and fluorinated gases would be expected to be significantly lower.

1.18 Model Limitations and Future Work

This analysis includes several limitations that should be addressed in future work. Greenhouse gas accounting is limited to CO_2 and does not include other greenhouse gases (e.g., CH_4, N_2O) or CO_2-equivalent metrics, which may be relevant for certain processes and supply chains. As more data is available on plant uptake rates the remediation timeframes and specific biomass characteristics relevant to biochar production and upkeep/matenance should be updated. Additionally, work on leaching behavior should be considered with the change of pH within these systems and the impact that has on other parameters such as soil organic carbon content.

In addition, the economic framework is simplified and does not fully capture key drivers of project feasibility, including financing structure, labor requirements, and capital costs that vary with system scale and configuration. This analysis also does not account for the added cost of soil and biochar characterization of PFAS which would be required. Finally, a more rigorous techno-economic analysis, including site-specific cost assumptions, is needed to better constrain the economic viability and scalability of the proposed approach.Uncertainty in PFAS partitioning and plant uptake also represents a key limitation. The modeling framework relies on pH-dependent Kd relationships and empirically derived uptake functions; however, PFAS sorption and bioavailability are influenced by multiple interacting soil properties, including organic carbon content, mineralogy, ionic strength, and competing solutes, as highlighted in prior studies and meta-analyses(*46*, *47*). As a result, the relationship between pH, mobility, and plant uptake used here should be interpreted as a simplified representation of a more complex system, and site-specific variability may lead to different outcomes. As more data become available on plant

uptake rates and compound-specific behavior, remediation timeframes and biomass characteristics relevant to biochar production and management should be refined.

Additionally, changes in pH may influence PFAS leaching behavior and broader soil chemistry, including interactions with soil organic carbon, which are not fully captured in the current framework and warrant further investigation. This analysis focuses on two long-chain PFAAs (PFOS and PFOA); however, extending this framework to additional PFAS, including compounds such as TFA with different mobility and plant uptake behavior, will be important as regulatory and scientific understanding evolves.

2.0 FIGURES

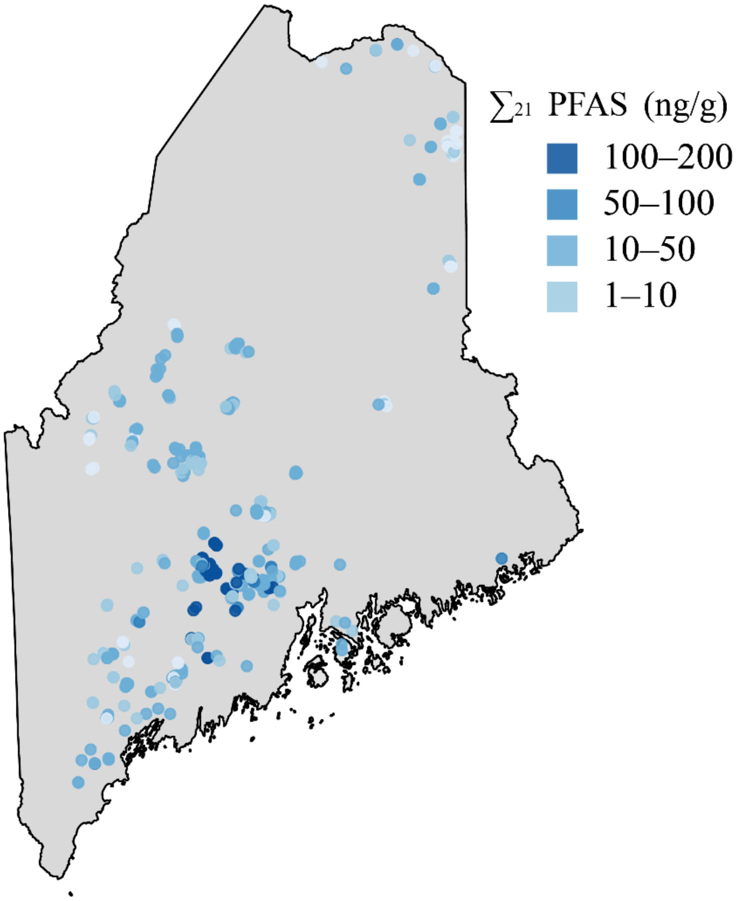

Fig S1: Distribution of impacted farmland across the state of Maine and total $\sum_{21}$ PFAS concentrations at the different sites tested (n= 563)

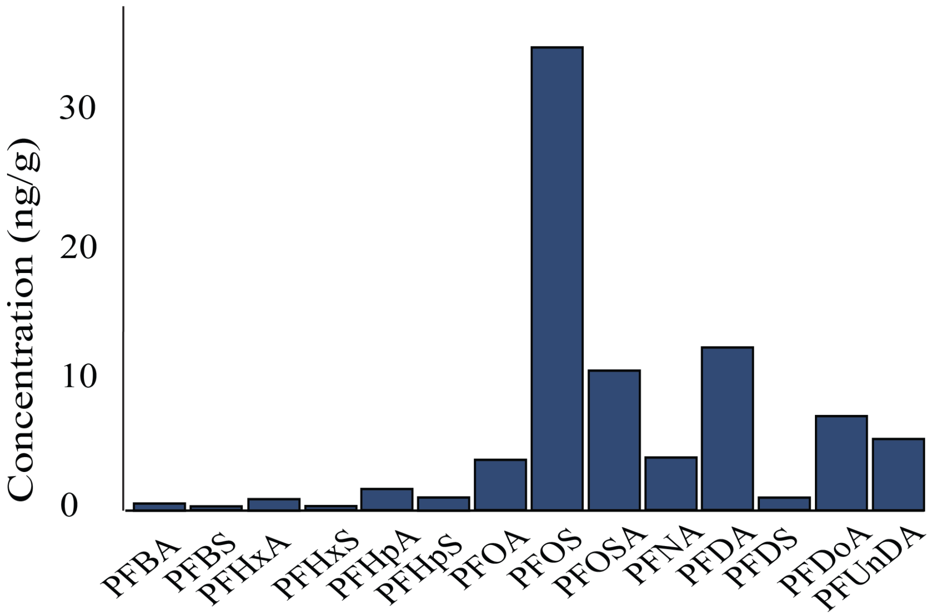

Fig S2. Concentration of 21 PFAS across the Maine DEP dataset (n=563)

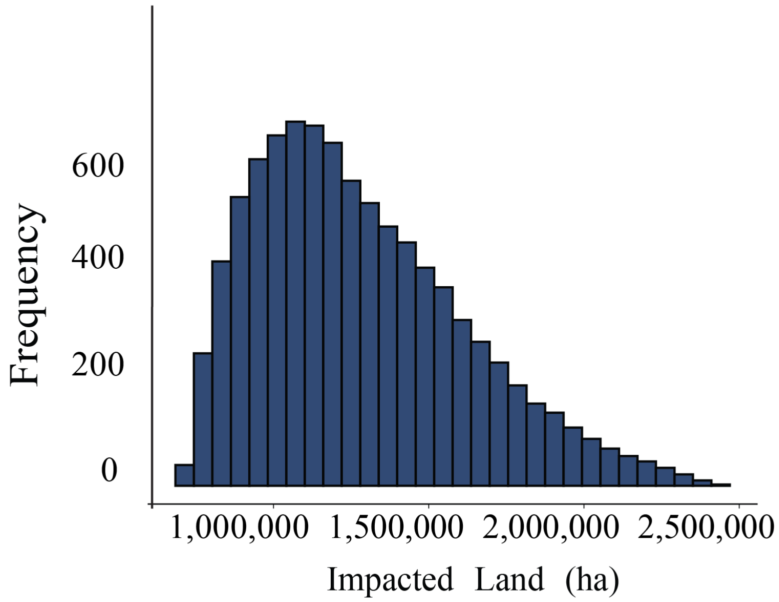

Fig S3. Probability distribution of the estimated total area of PFAS-impacted land in the United States.

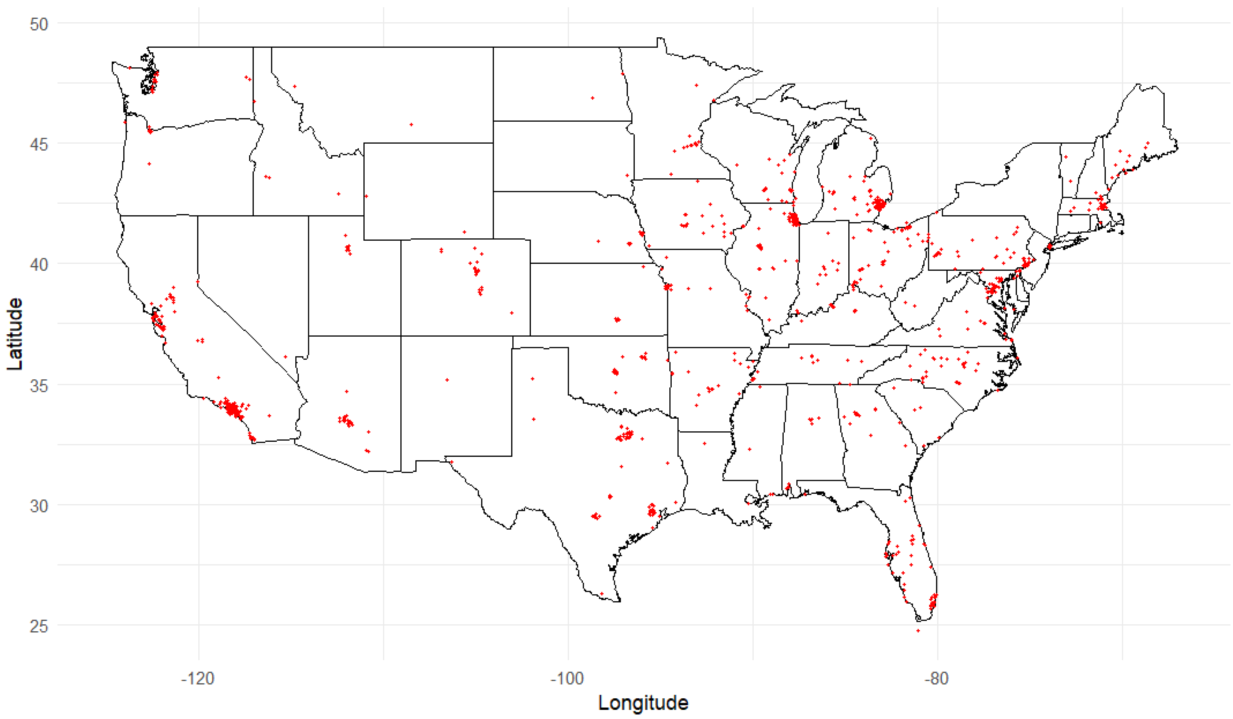

Fig S4. Spatial distribution of 1,000 hectare plots of PFAS impacted Agricultural land across the U.S.

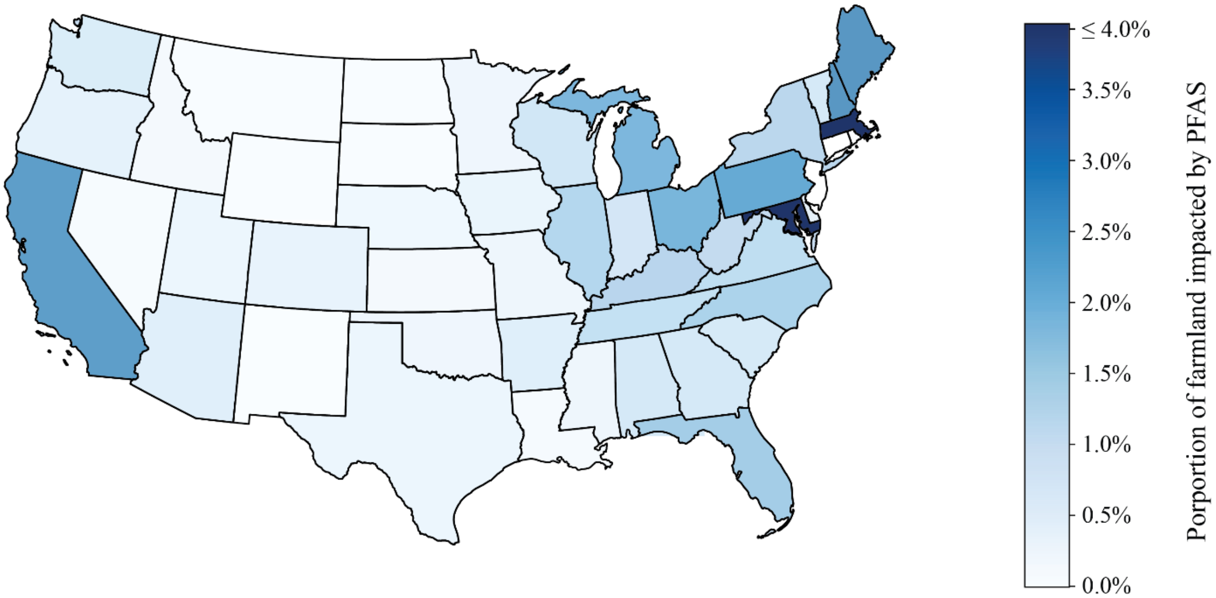

Fig S5. Proportion of the total farmland within each state that is impacted by PFAS.

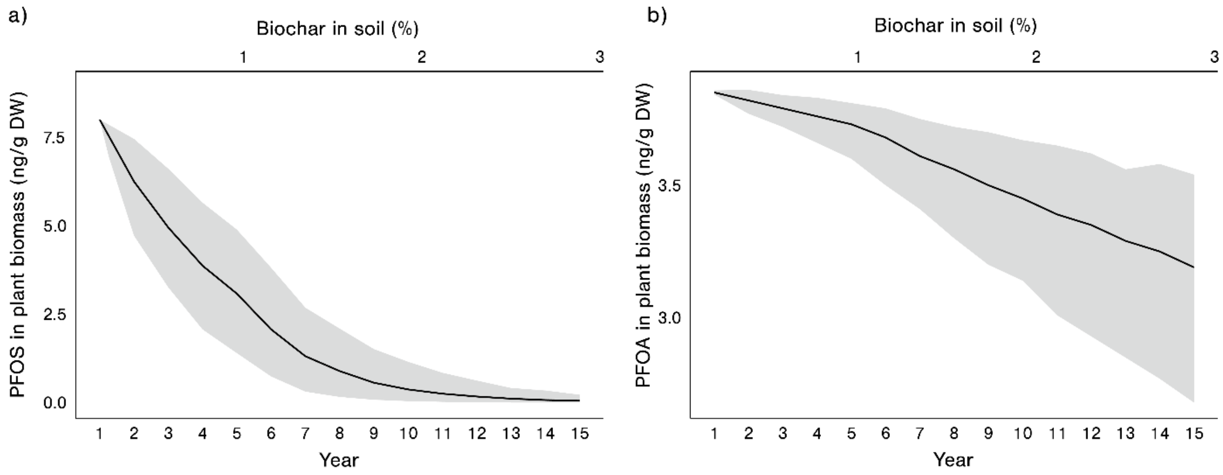

Fig S6. Concentrations of (a) PFOS and (b) PFOA in plant tissue over time as increasing amounts of biochar are amended to soils. The grey shaded region indicates the 90% uncertainty interval.

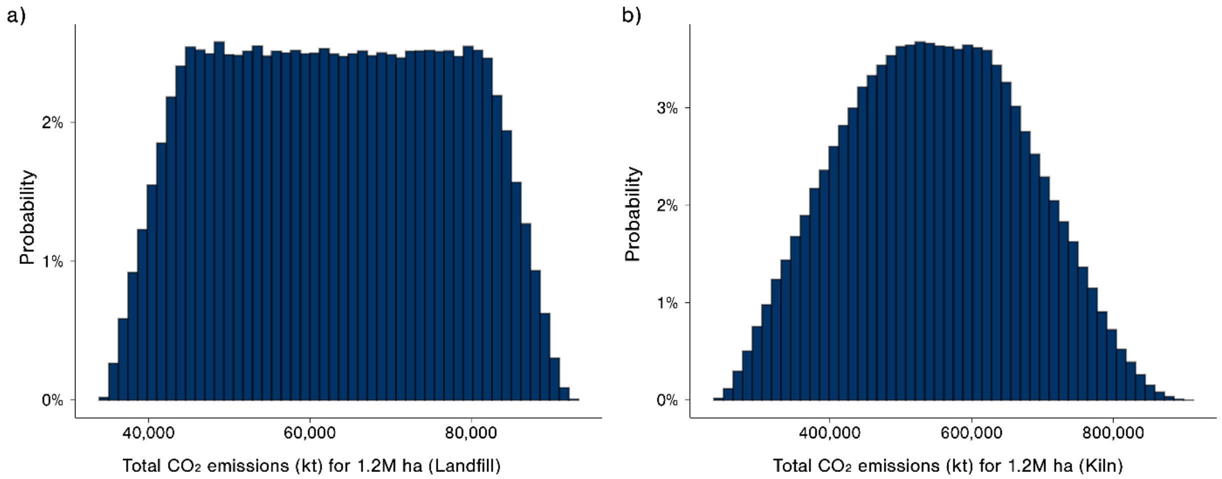

Fig S7. Probability distributions for total CO_2 emissions associated with remediating 1.2 million hectares of PFAS-impacted land.a) Landfill disposal scenario and b*)* Kiln incineration scenario. Bars represent Monte Carlo outcomes plotted as probability distributions

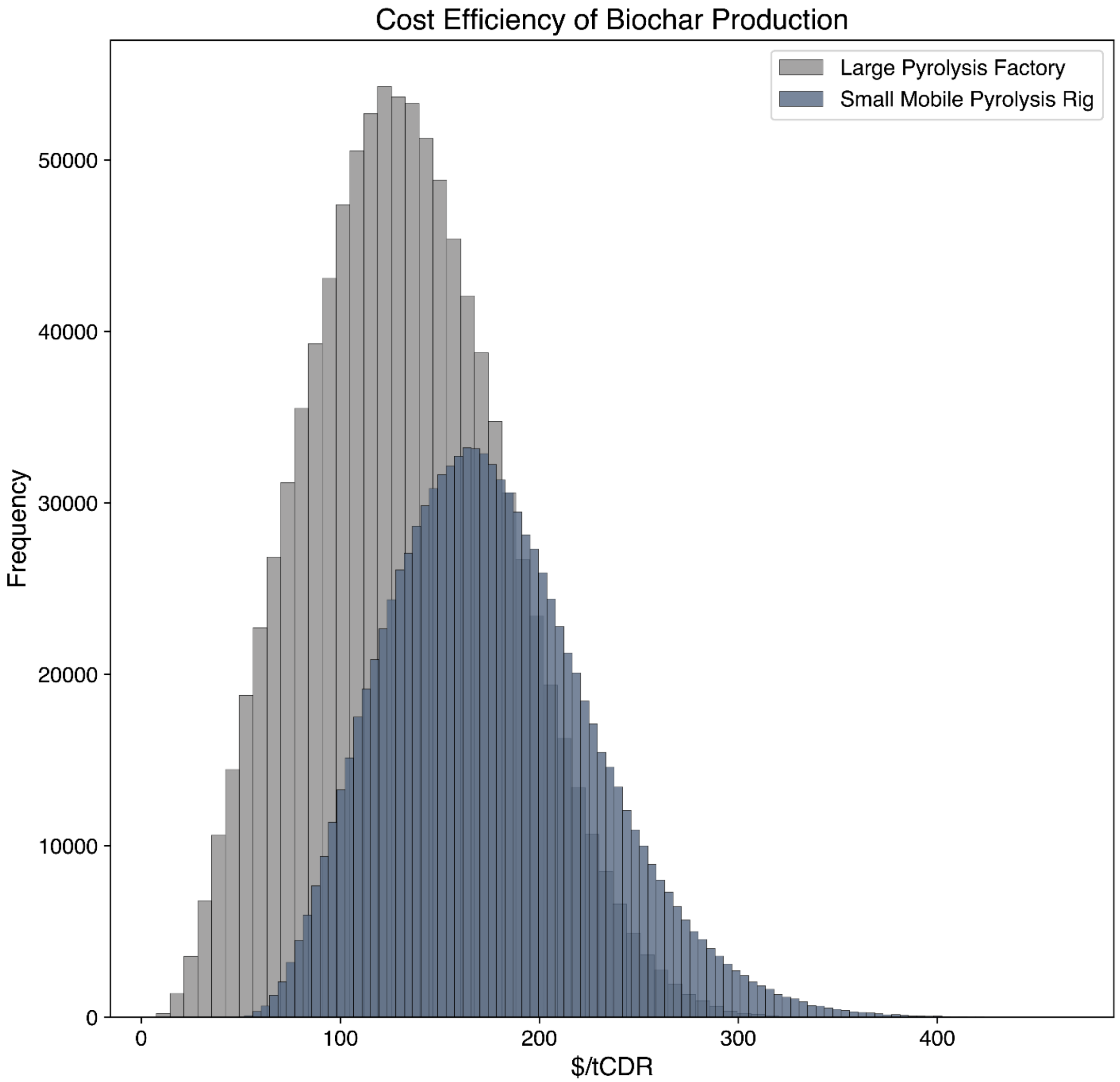

Fig S8. Results of the cost per ton of CDR between large scale pyrolysis or small transportable units

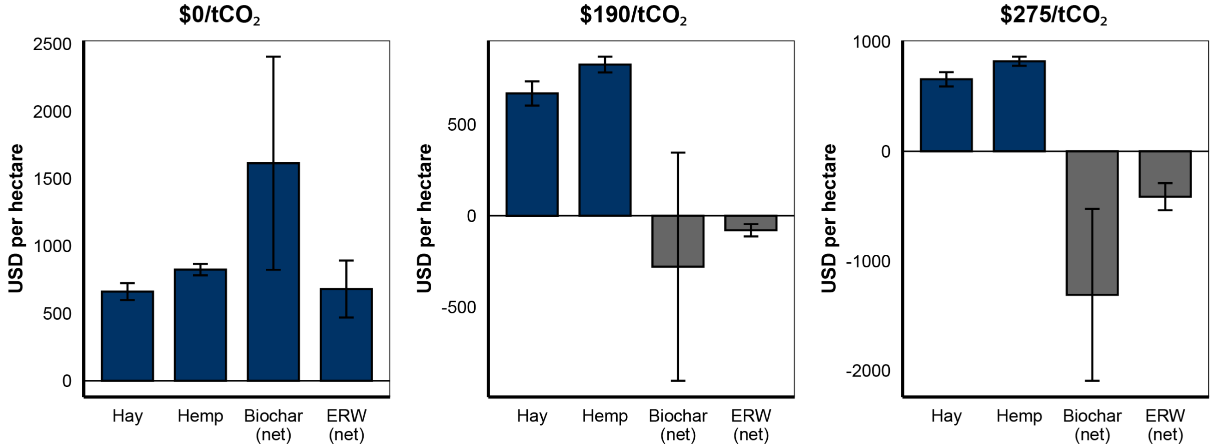

Fig S9. Cost breakdown of the major components of deployment of the remediation strategy as the carbon price changes. Error bars represent one standard deviation in estimates.

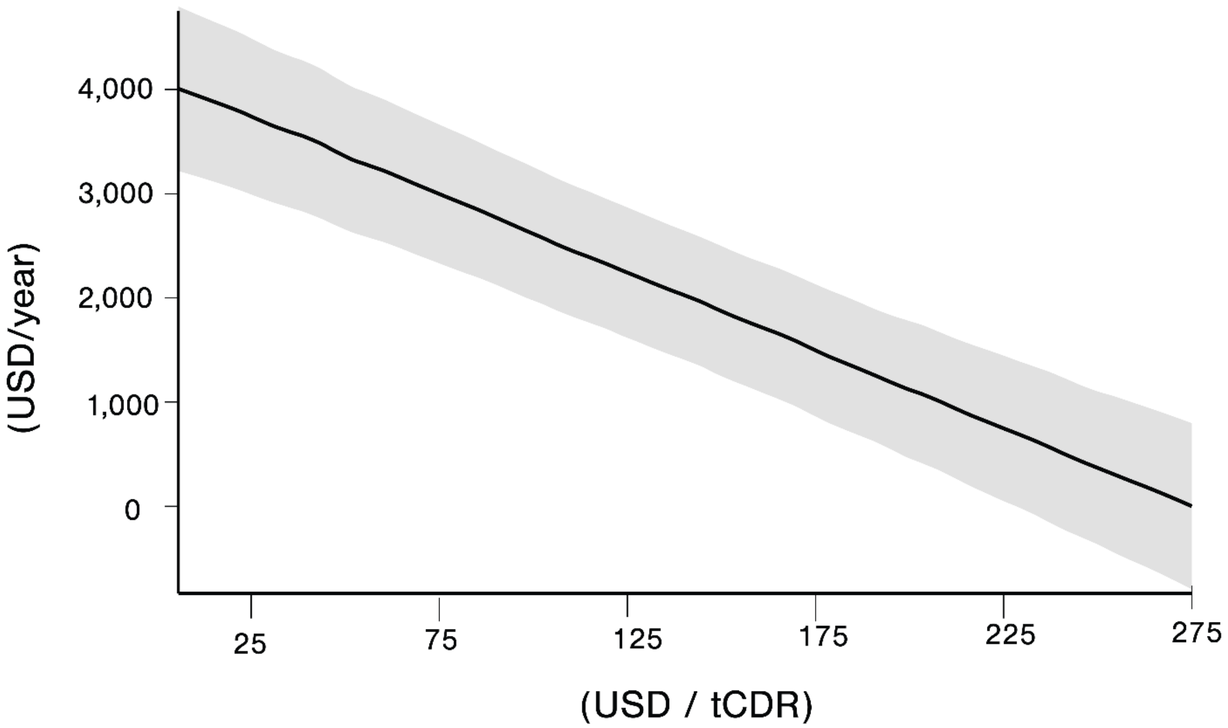

Fig S10. Break-even analysis showing the annual remediation cost per hectare as a function of the carbon dioxide removal (CDR) sale price. The gray shaded region denotes the 90% confidence interval.

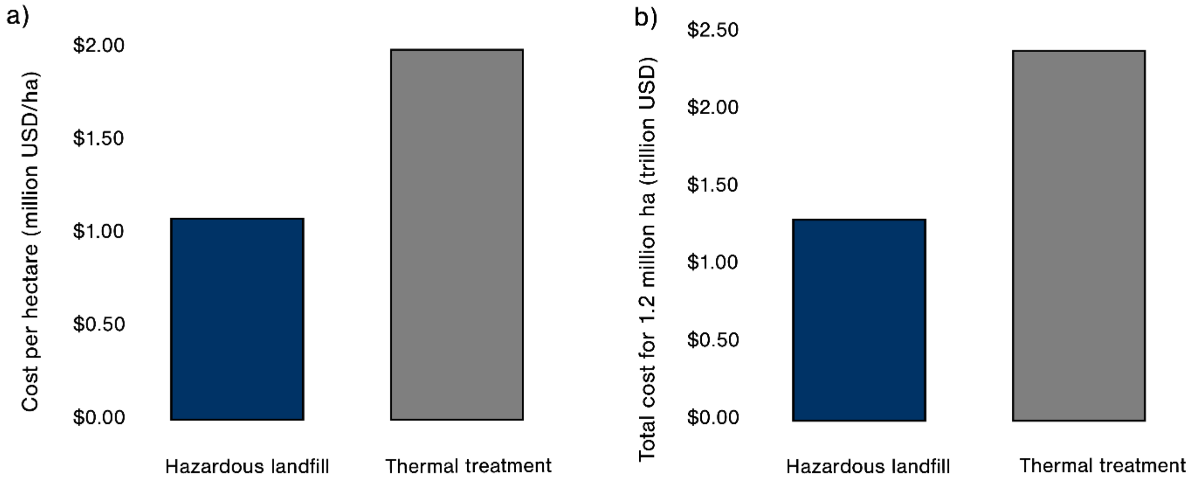

Fig S11**.** Estimated cost of conventional remediation for PFAS-impacted soils across agricultural lands. A) shows the estimated cost per hectare for excavation and disposal of contaminated soil via hazardous-waste landfill or thermal treatment. b) shows the corresponding total national cost assuming 1.2 million hectares of impacted land

3.0 TABLES

Table S1. Parameters, values, and sources for the calculation of the amount of PFAS impacted land in the U.S. from the historic application of biosolids used in equations 1-2

Parameter	Symbol	Description	Range/Value	Units	Source/Notes
Application rate	application rate	Annual biosolids application rate per hectare	6.93 – 11.03	metric tons/hectare/year	(*8*, *48*)
Duration of application	duration	Number of years biosolids were applied	7 – 15	years	(*8*, *48*)
Total application per hectare	B_h	Total applied biosolids per hectare over full application period	Derived	metric tons/hectare	Calculated
Number of simulations	n	Total Monte Carlo iterations	100,000	—	—
Cumulative biosolids applied	C_b	Total dry biosolids applied from 1970 - 2018	118,950,000	metric tons	Calculated
U.S. agricultural land area	—	Reference value for percent of total U.S. ag land impacted	175,619,491	hectares	(*10*)

Table S2. Site Condition Parameters and pH-Dependent K_{oc} Values Used in PFAS Remediation

Parameter	Symbol	Description	Range/Value	Units	Source/Notes
Initial organic carbon partition coefficient	K_{oc}, Year 0	Baseline soil organic carbon partition coefficient for PFAS	PFOS (900-1900)<br>PFOA (250-450)	L/kg	(*16–19*)
Soil pH	pH	Soil acidity	4.5 – 6.5	Unitless	(*49*)
Fraction of organic carbon	f_{oc}	Fraction of organic carbon in soil	0.01 – 0.03	Unitless	(*50*)
Distribution coefficient	K_d	Partition coefficient between soil and water	Compound and pH-dependent	L/kg	Calculated
Maximum sorption	K_{dmax}	Maximum Kd used to scale decay based on upper limits	28 PFOA<br>231 PFOS	L/kg	(*19*)
Minimum uptake efficiency	min_{eff}	Minimum plant uptake fraction at highest Kd	0.01 PFOA<br>0.005 PFOS	unitless	Estimated Lower bound for plant uptake
Reference growing season length	G_{ref}	Duration of the baseline growing season	90	days	(*15*)
Sampled growing season length	G_s	Simulated annual growing season length	90 – 220	days	(*51*)
PFAS soil concentration	C_{soil}, Year 0	Initial soil concentration of PFAS	Median values across sites	ng/g or mg/kg	(*9*)
Hemp PFOS removal	$R_{Hemp\ 0}$	Initial removal rate of PFOS	0.53	Percent removal	(*15*)
Hemp PFOA removal	$R_{Hemp\ 0}$	Initial removal rate of PFOA	1.3	Percent removal	(*15*)
Fescue PFOS removal	$R_{Fescue\ 0}$	Initial removal rate of PFOS	3.8	Percent removal	(*21*)
Fescue PFOA removal	$R_{Fescue\ 0}$	Initial removal rate of PFOA	4.9	Percent removal	(*21*)

Table S3. Parameters of variables used to calculate biochar competitive sorption effects in equations 7-11

Parameter	Symbol	Description	Range/Value	Units	Source/Notes
Number of simulations	n	Total Monte Carlo iterations	1,000	—	—
Simulation duration	years	Total number of years modeled	20	years	—
Mean annual biochar mass	$Mass_{biochar}$	Average biochar applied each year	7.5	metric tons / ha	Calculated
SD of annual biochar mass	$Mass_{biochar,sd}$	Standard deviation of annual biochar mass	1.0	metric tons / ha	Calculated
Soil mass per hectare	Mass soil	Mass of soil in one hectare of land	3,900	metric tons	(*23*)
Organic carbon fraction	f_{oc}	Soil organic carbon content	0.02	unitless	(*23*)
Biochar-water partitioning coefficient PFOS	$K_{f\ PFOS}$	Sorption coefficient of PFOS on biochar	$10^{4.8}$	L/kg	(*43*, *52*, *53*)
Biochar-water partitioning coefficient PFOA	$K_{f\ PFOA}$	Sorption coefficient of PFOA on biochar	$10^{3.3}$	L/kg	(*43*, *52*, *53*)
Log-SD of Kd (PFOS/PFOA)	$\sigma_{log\ Kd}$	Standard deviation on log-scale for Kd sampling	0.10/0.15	L/kg	(*43*, *52*, *53*)
Initial PFOS soil concentration	$C_{soilPFOS}$	PFOS concentration in Year 0 soil	100	ng/g	Representing >90 percentile
Initial PFOA soil concentration	$C_{soilPFOA}$	PFOA concentration in Year 0 soil	4	ng/g	Average site conditions

Table S4. Summary table for literature results of biochar addition to soils and plant uptake dynamic or leaching behavior.

Biochar	Plant	Soil Type	Application Rate	PFAS/ PFOS Uptake	Source
Black Owl Biochar Environmental Ultra™	Grass-Legume (9 varieties)	Silt loam, Silty Clay Loam	0%, 0.05%, 0.2%, 1%	Stabilized at 1% biochar. Increased uptake at low doses (0.05%)	Ilango et. al, 2024(*28*)
Organic custom wood and grass derived	Grass-Legume (9 varieties)	Silt Loam, Silty clay loam	0%, 0.05%, 0.2%, 1%	Stabilized at 1% biochar	Ilango et. al, 2024(*28*)
Luna s.r.l	Tomato (*Lycopersicum esculentum Mill., cv. Roma*)	Clay Loam	3%*	Reduction in leaves (-45% - -84%), fruits -61%.	Battisti et. al, 2024(*26*)
Luna s.r.l	Red Chicory (*Cychorium intybus L., cv.* late Treviso)	Clay Loam	3%*	Reduction in leaves (-74%), increase in soil for all PFAS.	Battisti et. al, 2024(*26*)
Forest Wood Waste	Timothy-grass	Sandy loam	0.2% , 2%	Increased plant uptake by 120% in 0.2% amended soil. Decreased leachable PFAS from soil.	Zhang et al 2022(*27*)
Granular activated carbon	Timothy-grass	Sandy loam	0.2% , 2%	Total PFAS concentration in grass shoots was 2.77% of grass from non-amended soil.	Zhang et al 2022(*27*)
Waste-Based (clean wood chips, , activated waste timber, digested sewage and dewatered raw sewage)	-	Sandy	1%	PFOS leachate concentration reduced by 99.9% with activated waste timber biochar at 1%.	Sørmo et. al, 2024(*24*)
Pine	-	Loamy Sand, Sandy Clay Loam	0-5%	Effect higher in sandy clay loam (sorption 69.8±4.9%) than loamy sand (sorption 11±4.5%)	
Waste timber	-	Moraine	5%	98-100% reduction in leachate concentration in low TOC soil, 23-100% reduction for high TOC.	Sørmo et. al, 2021

* Calculated with an estimated soil density = 1300 kg/m^3

Table S5. Parameters, values, and sources for the calculation of the amount of concentration of PFOS in pasture grass which meets risk based thresholds

**Parameter**	**Description**	**Value**	**Units**	**Source**
C_{soil}	Soil screening level (hay-based farm scenario)	6,800	ng/kg dry soil	(*54*)
TF_{plant}	Soil-to-plant transfer factor (hay)	0.07	unitless	(*54*, *55*)
MLF	Soil mass loading factor (soil on hay surface)	0.034	g dry soil / g dry plant	(*29*)
$\mathrm{C_{plant}}$	Back-calculated plant concentration (threshold)	707	ng/kg dry plant	Calculated

Table S6. Values for biomass yields and conversations of biomass to biochar

Parameter	Symbol	Distribution	Range	Units	Source
Biomass yield	Y_b	Linear with latitude	10–18	tons ha^{-1} yr^{-1}	(*56*)
Biochar conversion efficiency	η	Uniform	0.27–0.32	dimensionless	(*57*)
Biochar C content	C_f	Uniform	0.60–0.80	dimensionless	(*58*)
Area grid cell	A	Determined	1,000	ha	-
Pyrolysis emission rate	ϵ_{pyro}	Uniform	20-60	kg CO_2 per ton of biomass	(*59*, *60*)

Table S7. The parameters, values, and sources for the calculation of emissions from the traditional remediation of PFAS impacted land

Parameter	Symbol	Description	Range	Units	Source/Notes
Full Capacity Fuel Efficiency Range	F_F	Efficiency of diesel fuel for a full semi truck	8.73355 – 10.919	km/gal	(*35*)
Diesel Emissions	E_d	Emissions from diesel fuel.	0.01018	t/gal	(*61*)
Truck Load	L_T	Maximum load of semi truck	23 – 24	t	(*62*)
Landfill Distance	d_l	Distance in km to the nearest landfill for hazardous material.	322 – 805	km	Estimated
Rotary Kiln Distance	d_k	Distance in km to the nearest rotary kiln.	362 – 402	km	Estimated
Soil Depth	s_d	Average soil depth of contamination removal.	0.30	m	(*63*)
Soil Density	ρ_s	Average soil density.	1.3	t/m^3	(*23*)
Rail emission factor	E_r	CO_2 emissions per ton-km (rail)	2.1×10^{-5}	$tCO_2/(t\cdot km)$	(*64*)
Deadhead distance	d_d	Reposition distance for next job	16–64	km	Estimated
OM fraction in soil	f_{OM}	Organic matter mass fraction of soil	1–3.5%	%	(*65*)
C fraction of OM	$f_{C\|OM}$	Carbon fraction within OM	40–60%	%	(*66*, *67*)
Energy use per ton	ε_E	Kiln energy consumption	0.5–2.0	MMBtu/t	(*68*)
Rail switch threshold	d_s	Distance above which rail is used	120	km	Estimated
BTU Usage	u_{BTU}	Range of hourly BTU consumption of a rotary kiln	10-100	MMBtu/hr	(*69*)
Emissions per BTU	E_{BTU}	Emissions of CO_2 per million BTUs used during the operation of a rotary kiln.	116.65	Lbs CO_2/MMBtu	(*69*, *70*)
Kiln Processing Capacity	c_k	Average capacity of a kiln to pyrolyze contaminated soil.	22	t/hr	(*68*, *71*)
Average Farm Size	a_f	Average farm size in the United States	180	ha	(*72*)
Hazardous waste disposal cost	C_{lf}	Average price of soil disposal	277	USD / ton	(*39*)
Thermal treatment cost	C_{kn}	Average price of thermal treatment of soil	510	USD / ton	(*39*)

Table S8. Model parameters for Monte Carlo simulation of biochar cost

Parameter	Symbol	Description	Range	Units	Source/Notes
Consumption rate: large	B_L	Maximum hourly biomass consumption	2.5 – 4.5	t/hr	(*73*), quote estimates
Consumption rate: mobile	B_M	Maximum biomass capacity of mobile pyrolysis rig	1 – 2	t/hr	(*74*, *75*)
Operating hours: large	T_L	Hours of operation per day.	10 – 24	hr	Based on facility production estimates
Operating hours: mobile	T_M	Hours of operation per day.	6-10	hr	Based on labor
Biochar yeild	b_{BC}	Tons of biochar produced from one ton of biomass	0.27-0.32	-	(*74–76*)
Carbon content	C_{BC}	Fraction of carbon in biochar.	0.600 – 0.70	-	(*76*, *77*)
Biomass cost	P_B	Price of biomass per ton	0 – 70	$/t	(*78*)
Fuel efficiency range	F_F	Efficiency of diesel fuel for a full semi-truck	8.73355 – 10.919	km/gal	(*79*, *80*)
Average fuel consumption	F_M	Average fuel consumption of a mobile pyrolysis unit.	1.1	gal/hr	(*74*, *75*)
Diesel emissions	E_d	Emissions from diesel fuel.	0.01018	t/gal	(*81*)
Average fuel price	P_{fuel}	Average price of diesel fuel in USD.	3.67	$	(*82*)
Operating cost: large pyrolysis	O_L	Cost per hour of operating a large pyrolysis plant.	0-250	$/hr	Quote estimates.
Operating cost: mobile unit	O_M	Cost per hour of operating a mobile pyrolysis unit.	50	$/hr	Quote estimates.
Cost for mobile pyrolysis	P_M	Cost for the capital investment	200,000 – 360,000	$	Quote estimates.
Truck load	L_T	Maximum load of semi-truck	23 – 24	t	(*83*)
Driving distance	d_t	Distance in km material moved by truck.	50 – 200	km	Quote estimates
Trucking operating cost	P_{km}	Per-distance trucking operating cost	1.364 - 3.115	$/km	(*84*)

Preprocessing cost	P_{pre}	Cost for grinding/size reduction of biomass	5–10	$ t^{-1}	Quote estimates
Storage fraction	$f_{storage}$	Fraction of biomass requiring storage	0.2–0.5	—%	Quote estimates
Storage cost	$P_{storage}$	Cost of short-term storage and handling of biomass	4–16	$ t^{-1} s	Quote estimates
Storage loss	$L_{storage}$	Fractional dry matter loss during storage	0.01–0.03	—%	(*74–76*)
Ash fraction	f_{ash}	Fraction of biomass converted to residual solids/ash	0.01–0.02	—%	(*74–76*)
Ash disposal	P_{ash}	Cost of handling/disposal of residual solids	40–80	$ t^{-1}	Quote estimates

Table S9. Parameters, values, and sources for the economic costs analysis

Parameter	Symbol	Range / Mean ± SD	Units	Source
Simulation years	T	22.5 ± 4.5	yr	Calculated
Fescue production cost	C_{fescue}	550–780	USD ha^{-1} yr^{-1}	(*85*, *86*)
Hemp production cost	C_{hemp}	750–900	USD ha^{-1} yr^{-1}	(*87*)
Farmer income offset	I_{farmer}	300–500	USD ha^{-1} yr^{-1}	Estimated
Biochar CDR rate	$Q_{biochar}$	4.8–16.5	tCO_2 ha^{-1} yr^{-1}	Calculated
ERW CDR rate	Q_{ERW}	2–6	tCO_2 ha^{-1} yr^{-1}	(*33*, *88*)
Biochar cost rate	$P_{biochar}$	160 ± 70	USD tCO_2^{-1}	Calculated
ERW cost rate	P_{ERW}	160–180	USD tCO_2^{-1}	(*33*)
Biochar sale price	$S_{biochar}$	125–175	USD tCO_2^{-1}	(*89*)
ERW sale price	S_{ERW}	300–325	USD tCO_2^{-1}	(*89*)
Social Cost Carbon	SCC	190-275	USD tCO_2^{-1}	(*90*)

References

1. Marc Mills, PFAS Treatment in Biosolids – State of the Science (2020). https://www.epa.gov/sites/default/files/2020-10/documents/r1-pfas_webinar_day_2_session_6_mills_final.pdf.
2. N. Beecher, Greg, Kester, Nora, Goldstein, Maile Lono-Batura, Juliana, Beecher, Janine, Burke-Wells, B. Toffey, National Summary, *National Biosolids Data State Summaries* (2022). https://www.biosolidsdata.org/national-summary.
3. G. Munoz, A. M. Michaud, M. Liu, S. Vo Duy, D. Montenach, C. Resseguier, F. Watteau, V. Sappin-Didier, F. Feder, T. Morvan, S. Houot, M. Desrosiers, J. Liu, S. Sauvé, Target and Nontarget Screening of PFAS in Biosolids, Composts, and Other Organic Waste Products for Land Application in France. *Environ. Sci. Technol.*, doi: 10.1021/acs.est.1c03697 (2021).
4. O. US EPA, Basic Information about Sewage Sludge and Biosolids (2024). https://www.epa.gov/biosolids/basic-information-about-sewage-sludge-and-biosolids.
5. N. Beecher, Greg, Kester, Nora, Goldstein, Maile Lono-Batura, Juliana, Beecher, Janine, Burke-Wells, B. Toffey, State Summaries, *National Biosolids Data State Summaries* (2022). https://www.biosolidsdata.org/state-summaries.
6. S. G. Hughes, “PFAS in Biosolids: A Review of State Efforts & Opportunities for Action” (2023).
7. G. R. Johnson, PFAS in soil and groundwater following historical land application of biosolids. *Water Res.* **211**, 118035 (2022).
8. U.S. EPA, “Biosolids Technology Fact Sheet: Land Application of Biosolids” (EPA 832-F-00-064, Office of Water, Washington, DC, 2000); https://www.epa.gov/sites/default/files/2018-11/documents/land-application-biosolids-factsheet.pdf.
9. Maine DEP, Maine DEP PFAS Investigation (Formerly the “Septage and Sludge Map”) (2024). https://maine.maps.arcgis.com/apps/webappviewer/index.html?id=468a9f7ddcd54309bc1ae8ba173965c7.
10. USDA, Farms and Land in Farms 2024 Summary. (2024).
11. Richard Becker, Allan R. Wilks, raw Geographical Maps, version R package version 3.4.2 (2023); https://CRAN.R-project.org/package=maps.

12. U. N. H. S. Programme, *Global Atlas of Excreta, Wastewater Sludge, and Biosolids Management: Moving Forward the Sustainable and Welcome Uses of a Global Resource* (UN-HABITAT, 2009).

13. U.S. Census Bureau., Gazetteer Files - State, (2021); https://www.census.gov/geographies/reference-files/time-series/geo/gazetteer-files.html.

14. B. Woollen, C. Schnabel, S. Zhang, C. T. Reinhard, T. J. Suhrhoff, B. Buma, N. Planavsky, "Estimating Soil pH on Agricultural Lands as a Constraint on Enhanced Weathering Potential" (2024; https://ui.adsabs.harvard.edu/abs/2024AGUFMGC13Q0427W)vol. 2024, pp. GC13Q-0427.

15. W. Nassazzi, T.-C. Wu, J. Jass, F. Y. Lai, L. Ahrens, Phytoextraction of per- and polyfluoroalkyl substances (PFAS) and the influence of supplements on the performance of short–rotation crops. *Environ. Pollut.* **333**, 122038 (2023).

16. H. Campos-Pereira, Dan B. Kleja, L. Ahrens, A. Enell, J. Kikuchi, M. Pettersson, J. P. Gustafsson, Effect of pH, surface charge and soil properties on the solid–solution partitioning of perfluoroalkyl substances (PFASs) in a wide range of temperate soils. *Chemosphere* **321**, 138133 (2023).

17. X. Chen, L. Zhu, X. Pan, S. Fang, Y. Zhang, L. Yang, Isomeric specific partitioning behaviors of perfluoroalkyl substances in water dissolved phase, suspended particulate matters and sediments in Liao River Basin and Taihu Lake, China. *Water Res.* **80**, 235–244 (2015).

18. R. Hunter Anderson, D. T. Adamson, H. F. Stroo, Partitioning of poly- and perfluoroalkyl substances from soil to groundwater within aqueous film-forming foam source zones. *J. Contam. Hydrol.* **220**, 59–65 (2019).

19. C. P. Higgins, R. G. Luthy, Sorption of Perfluorinated Surfactants on Sediments. *Environ. Sci. Technol.* **40**, 7251–7256 (2006).

20. I. Ross, J. McDonough, J. Miles, P. Storch, P. T. Kochunarayanan, E. Kalve, J. Hurst, S. S. Dasgupta, J. Burdick, A review of emerging technologies for remediation of PFASs. *Remediat. J.* **28**, 101–126 (2018).

21. D. K. Huff, L. A. Morris, L. Sutter, J. Costanza, K. D. Pennell, Accumulation of six PFAS compounds by woody and herbaceous plants: potential for phytoextraction. *Int. J. Phytoremediation* **22**, 1538–1550 (2020).

22. J.-J. Ortega-Calvo, J. Harmsen, J. R. Parsons, K. T. Semple, M. D. Aitken, C. Ajao, C. Eadsforth, M. Galay-Burgos, R. Naidu, R. Oliver, W. J. G. M. Peijnenburg, J. Römbke, G. Streck, B. Versonnen, From Bioavailability Science to Regulation of Organic Chemicals. *Environ. Sci. Technol.* **49**, 10255–10264 (2015).

23. Sumit Sharma, Jason Warren, Basics of Soil Bulk Density - Oklahoma State University (2024). https://extension.okstate.edu/fact-sheets/basics-of-soil-bulk-density.html.

24. E. Sørmo, C. B. M. Lade, J. Zhang, A. G. Asimakopoulos, G. W. Åsli, M. Hubert, A. I. Goranov, H. P. H. Arp, G. Cornelissen, Stabilization of PFAS-contaminated soil with sewage sludge- and wood-based biochar sorbents. *Sci. Total Environ.* **922**, 170971 (2024).

25. M. A. Holly, K. M. Gunn, D. Keymer, J. R. Sanford, Evaluation of Per- and Polyfluoroalkyl Substances Leaching from Biosolids and Mitigation Potential of Biochar through Undisturbed Soil Columns. *ACS EST Water* **4**, 413–426 (2024).

26. I. Battisti, A. R. Trentin, A. Sabia, A. Masi, G. Renella, Soil Amendment with Biochar Reduces the Uptake and Translocation of Perfluoroalkyl Substances by Horticultural Plants Grown in a Polluted Area. Social Science Research Network 4944118 [Preprint] (2024). https://doi.org/10.2139/ssrn.4944118.

27. W. Zhang, Y. Liang, Changing bioavailability of per- and polyfluoroalkyl substances (PFAS) to plant in biosolids amended soil through stabilization or mobilization. *Environ. Pollut.* **308**, 119724 (2022).

28. A. K. Ilango, W. Zhang, Y. Liang, Uptake of per- and polyfluoroalkyl substances by Conservation Reserve Program's seed mix in biosolids-amended soil. *Environ. Pollut.* **363**, 125235 (2024).

29. Maine DACF, Assessing PFAS Contamination and Managing Risks on Dairy Farms in Maine. (2022).

30. Y. Kanzaki, S. Zhang, N. J. Planavsky, C. T. Reinhard, Soil Cycles of Elements simulator for Predicting TERrestrial regulation of greenhouse gases: SCEPTER v0.9. *Geosci. Model Dev.* **15**, 4959–4990 (2022).

31. Y. Kanzaki, I. Chiaravalloti, S. Zhang, N. J. Planavsky, C. T. Reinhard, In silico calculation of soil pH by SCEPTER v1.0. *Geosci. Model Dev.* **17**, 4515–4532 (2024).

32. Y. Kanzaki, N. J. Planavsky, S. Zhang, J. Jordan, T. J. Suhrhoff, C. T. Reinhard, Soil cation storage is a key control on the carbon removal dynamics of enhanced weathering. *Environ. Res. Lett.* **20**, 074055 (2025).

33. D. J. Beerling, E. P. Kantzas, M. R. Lomas, P. Wade, R. M. Eufrasio, P. Renforth, B. Sarkar, M. G. Andrews, R. H. James, C. R. Pearce, J.-F. Mercure, H. Pollitt, P. B. Holden, N. R. Edwards, M. Khanna, L. Koh, S. Quegan, N. F. Pidgeon, I. A. Janssens, J. Hansen, S. A. Banwart, Potential for large-scale CO2 removal via enhanced rock weathering with croplands. *Nature* **583**, 242–248 (2020).

34. D. A. C. Manning, Enhanced rock weathering — A nature-based solution for climate mitigation. *Green Energy Sustain.* **5** (2025).

35. NACFE, NACFE: Fleet Fuel Study, *NACFE* (2024). http://nacfe.org/research/affs/.

36. US Army Corps of Engineers, "INLAND WATERWAYS TRANSPORT" (2018); https://www.mvp.usace.army.mil/Portals/57/docs/Navigation/Brochures/Inland_Waterway_Transport_Display_8-2-2018.pdf?ver=2018-08-15-154356-940#:~:text=Barges%20can%20move%20one%20ton,miles%20per%20gallon%20of%20fuel.

37. U.S. Congressional Budget Office, "Emissions of Carbon Dioxide in the Transportation Sector" (2022); https://doi.org/https://www.cbo.gov/system/files/2022-12/58566-co2-emissions-transportation.pdf.

38. D. Woolf, J. Lehmann, S. Ogle, A. W. Kishimoto-Mo, B. McConkey, J. Baldock, Greenhouse Gas Inventory Model for Biochar Additions to Soil. *Environ. Sci. Technol.* **55**, 14795–14805 (2021).

39. United States Chamber of Commerce, "PFOS and PFOA Private Cleanup Costs at Superfund Sites" (2022); https://www.uschamber.com/assets/documents/PFOS-and-PFOA-Private-Cleanup-Costs-at-Superfund-Sites-6.8.22.pdf.

40. B. Zhang, K. Lan, F. Yang, Y. Xu, D. Piotto, M. Ashton, Y. Yao, Innovative reforestation mosaics on marginal land in the globally important Mata Atlântica biome can create climate and economic co-benefits. *One Earth* **8** (2025).

41. N. Wu, K. Lan, Y. Yao, An integrated techno-economic and environmental assessment for carbon capture in hydrogen production by biomass gasification. *Resour. Conserv. Recycl.* **188**, 106693 (2023).

42. U.S. EPA, Final PFAS National Primary Drinking Water Regulation (2024). https://www.epa.gov/system/files/documents/2024-04/drinking-water-utilities-and-professionals-technical-overview-of-pfas-npdwr.pdf?utm_source=chatgpt.com.

43. B. Cantoni, A. Turolla, J. Wellmitz, A. S. Ruhl, M. Antonelli, Perfluoroalkyl substances (PFAS) adsorption in drinking water by granular activated carbon: Influence of activated carbon and PFAS characteristics. *Sci. Total Environ.* **795**, 148821 (2021).

44. N. Belkouteb, V. Franke, P. McCleaf, S. Köhler, L. Ahrens, Removal of per- and polyfluoroalkyl substances (PFASs) in a full-scale drinking water treatment plant: Long-term performance of granular activated carbon (GAC) and influence of flow-rate. *Water Res.* **182**, 115913 (2020).

45. IPCC, "Global-Warming-Potential-Values (August 2024)" (2024); https://ghgprotocol.org/sites/default/files/2024-08/Global-Warming-Potential-Values%20%28August%202024%29.pdf.

46. Y. Li, D. P. Oliver, R. S. Kookana, A critical analysis of published data to discern the role of soil and sediment properties in determining sorption of per and polyfluoroalkyl substances (PFASs). *Sci. Total Environ.* **628–629**, 110–120 (2018).

47. T. M. H. Nguyen, J. Bräunig, K. Thompson, J. Thompson, S. Kabiri, D. A. Navarro, R. S. Kookana, C. Grimison, C. M. Barnes, C. P. Higgins, M. J. McLaughlin, J. F. Mueller, Influences of Chemical Properties, Soil Properties, and Solution pH on Soil–Water Partitioning Coefficients of Per- and Polyfluoroalkyl Substances (PFASs). *Environ. Sci. Technol.* **54**, 15883–15892 (2020).

48. City of Fort Collins Water Reclamation, Biosolids Management Performance Report (2017). https://www.fcgov.com/utilities/img/site_specific/uploads/jw_09112018_biosolids-management-program-annual-report.pdf?utm_source=chatgpt.com.

49. G. Liu, E. Hanlon, Soil pH Range for Optimum Commercial Vegetable Production: HS1207/HS1207, 10/2012. *EDIS* **2012** (2012).

50. USDA, Soil Organic Matter (2014). https://www.nrcs.usda.gov/sites/default/files/2022-10/Soil%20Organic%20Matter.pdf?utm.com.

51. USDA, USDA Unveils Updated Plant Hardiness Zone Map : USDA ARS (2023). https://www.ars.usda.gov/news-events/news/research-news/2023/usda-unveils-updated-plant-hardiness-zone-map/?utm_source=chatgpt.com.

52. E. Sørmo, L. Silvani, N. Bjerkli, N. Hagemann, A. R. Zimmerman, S. E. Hale, C. B. Hansen, T. Hartnik, G. Cornelissen, Stabilization of PFAS-contaminated soil with activated biochar. *Sci. Total Environ.* **763**, 144034 (2021).

53. H. N. P. Vo, T. M. H. Nguyen, H. H. Ngo, W. Guo, P. Shukla, Biochar sorption of perfluoroalkyl substances (PFASs) in aqueous film-forming foams-impacted groundwater: Effects of PFASs properties and groundwater chemistry. *Chemosphere* **286**, 131622 (2022).

54. Maine CDC, Derivation of Action Levels for PFOS in Cows Milk (2017). https://www.maine.gov/dep/spills/topics/pfas/Derivation-of-Action-Levels-for-PFOS-in-Cows-Milk-03.28.17.pdf.

55. H. Yoo, J. W. Washington, T. M. Jenkins, J. J. Ellington, Quantitative Determination of Perfluorochemicals and Fluorotelomer Alcohols in Plants from Biosolid-Amended Fields using LC/MS/MS and GC/MS. *Environ. Sci. Technol.* **45**, 7985–7990 (2011).

56. S. P. Alesso, R. Tapias, J. Alaejos, M. Fernández, Biomass Yield and Economic, Energy and Carbon Balances of Ulmus pumila L., Robinia pseudoacacia L. and Populus × euroamericana (Dode) Guinier Short-Rotation Coppices on Degraded Lands under Mediterranean Climate. *Forests* **12**, 1337 (2021).

57. IPCC, Method for Estimating the Change in Mineral Soil Organic Carbon Stocks from Biochar Amendments (2019). https://www.ipcc-nggip.iges.or.jp/public/2019rf/pdf/4_Volume4/19R_V4_Ch02_Ap4_Biochar.pdf?utm_source=chatgpt.com.

58. S. Adhikari, E. Moon, J. Paz-Ferreiro, W. Timms, Comparative analysis of biochar carbon stability methods and implications for carbon credits. *Sci. Total Environ.* **914**, 169607 (2024).

59. Carbofex, “LCA Carbofex Biochar Report” (2022); https://carbofex.fi/wp-content/uploads/2023/05/Ecobio-LCA_Carbofex-Biochar-2023-v5.pdf.

60. J. E. Amonette, “Climate-focused Life Cycle Assessments of Biochar Production by an ARTi Pyrolysis Reactor and an Air Burners CharBoss® Air Curtain Incinerator” (2025); https://www.pnnl.gov/main/publications/external/technical_reports/PNNL-37486.pdf.

61. O. US EPA, Greenhouse Gas Emissions from a Typical Passenger Vehicle (2016). https://www.epa.gov/greenvehicles/greenhouse-gas-emissions-typical-passenger-vehicle.

62. U.S. DOT, Maximum Legal Vehicle Weight Limits (2024). https://www.dot.nh.gov/sites/g/files/ehbemt811/files/inline-documents/truck-weights.pdf.

63. R. Alvarez-Ruiz, L. S. Lee, Y. Choi, Fate of per- and polyfluoroalkyl substances at a 40-year dedicated municipal biosolids land disposal site. *Sci. Total Environ.* **954**, 176540 (2024).

64. Office of Rail and Road, Rail Emissions. https://dataportal.orr.gov.uk/media/1993/rail-emissions-2020-21.pdf?utm_source=chatgpt.com.

65. Vern Grubinger, Managing Nitrogen on Organic Farms (2005). https://www.uvm.edu/vtvegandberry/factsheets/managingNorganic.html?utm_source=chatgpt.com.

66. D. W. Pribyl, A critical review of the conventional SOC to SOM conversion factor. *Geoderma* **156**, 75–83 (2010).

67. A. J. Franzluebbers, Soil organic matter stratification ratio as an indicator of soil quality. *Soil Tillage Res.* **66**, 95–106 (2002).

68. US EPA, Incinerator and Cement Kiln Capacity For Hazardous Waste Treatment (1986). https://nepis.epa.gov/Exe/ZyNET.exe/2000TL9E.txt?ZyActionD=ZyDocument&Client=EPA&Index=1986%20Thru%201990&Docs=&Query=&Time=&EndTime=&SearchMethod=1&TocRestrict=n&Toc=&TocEntry=&QField=&QFieldYear=&QFieldMonth=&QFieldDay=&UseQField=&IntQFieldOp=0&ExtQFieldOp=0&XmlQuery=&File=D%3A%5CZYFILES%5CINDEX%20DATA%5C86THRU90%5CTXT%5C00000009%5C2000TL9E.txt&User=anonymous&Password=anonymous&SortMethod=h%7C-&MaximumDocuments=1&FuzzyDegree=0&ImageQuality=r75g8/r75g8/x150y150g16/i425&Display=hpfr&DefSeekPage=&SearchBack=ZyActionL&Back=ZyActionS&BackDesc=Results%20page&MaximumPages=1&ZyEntry=3.

69. S. A. Ishak, H. Hashim, Low carbon measures for cement plant – a review. *J. Clean. Prod.* **103**, 260–274 (2015).

70. IPCC, Emissions From Waste Incineration (2018). https://www.ipcc-nggip.iges.or.jp/public/gp/bgp/5_3_Waste_Incineration.pdf.

71. J. Han, C. Xu, J. Jin, J. Hu, PCNs, PCBs, and PCDD/Fs in Soil around a Cement Kiln Co-Processing Municipal Wastes in Northwestern China: Levels, Distribution, and Potential Human Health Risks. *Int. J. Environ. Res. Public. Health* **19**, 12860 (2022).

72. USDA, 2022 Census of Agriculture: Number of U.S. farms falls below 2 million | Economic Research Service (2022). https://www.ers.usda.gov/data-products/charts-of-note/chart-detail?chartId=108629.

73. F.-X. Collard, S. Wijeyekoon, P. Bennett, Commercial status of direct thermochemical liquefaction technologies.

74. ARTi - Pyrolysis Reactors, *ARTi* (2025). https://www.arti.com/reactors/.

75. CharBoss (2023). https://airburners.com/products/boss-series/charboss/.

76. F. Amalina, A. S. A. Razak, S. Krishnan, H. Sulaiman, A. W. Zularisam, M. Nasrullah, Biochar production techniques utilizing biomass waste-derived materials and environmental applications – A review. *J. Hazard. Mater. Adv.* **7**, 100134 (2022).

77. M. Coleman, D. Page-Dumroese, J. Archuleta, P. Badger, W. Chung, T. Venn, D. Loeffler, G. Jones, K. McElligott, Can portable pyrolysis units make biomass utilization affordable while using bio-char to enhance soil productivity and sequester carbon? *Jain Theresa B Graham Russell T Sandquist Jonathan Integr. Manag. Carbon Sequestration Biomass Util. Oppor. Chang. Clim. Proc. 2009 Natl. Silvic. Workshop 2009 June 15-18 Boise ID Proc. RMRS-P-61 Fort Collins CO US Dep. Agric. For. Serv. Rocky Mt. Res. Stn. P 159-168* **61**, 159–168 (2010).

78. C. Hellwinckel, D. Ugarte, J. Field, M. Langholtz, Chapter 5: Biomass from Agriculture, Oak Ridge National Laboratory (ORNL), Oak Ridge, TN (United States), Bioenergy Knowledge Discovery Framework (BEKDF) (2024); https://doi.org/10.23720/BT2023/2316171.

79. Hale, 10 Tips to Managing Semi-Truck Fuel Efficiency, *Hale Trailer Brake & Wheel* (2025). https://haletrailer.com/blog/semi-truck-fuel-efficiency/.

80. Fuel Economy of Heavy Duty Vehicles (1976). https://nepis.epa.gov/Exe/ZyNET.exe/9100UPS2.TXT?ZyActionD=ZyDocument&Client=EPA&Index=1976+Thru+1980&Docs=&Query=&Time=&EndTime=&SearchMethod=1&TocRestrict=n&Toc=&TocEntry=&QField=&QFieldYear=&QFieldMonth=&QFieldDay=&IntQFieldOp=0&ExtQFieldOp=0&XmlQuery=&File=D%3A%5Czyfiles%5CIndex%20Data%5C76thru80%5CTxt%5C00000019%5C9100UPS2.txt&User=ANONYMOUS&Password=anonymous&SortMethod=h%7C-&MaximumDocuments=1&FuzzyDegree=0&ImageQuality=r75g8/r75g8/x150y150g16/i425&Display=hpfr&DefSeekPage=x&SearchBack=ZyActionL&Back=ZyActionS&BackDesc=Results%20page&MaximumPages=1&ZyEntry=1&SeekPage=x&ZyPURL.

81. O. US EPA, Greenhouse Gas Emissions from a Typical Passenger Vehicle (2016). https://www.epa.gov/greenvehicles/greenhouse-gas-emissions-typical-passenger-vehicle.

82. US EIA, Gasoline and Diesel Fuel Update. https://www.eia.gov/petroleum/gasdiesel/index.php.

83. NH DOT, Transport Trucking Weight Limits (1992). https://www.dot.nh.gov/sites/g/files/ehbemt811/files/inline-documents/truck-weights.pdf.

84. Bureau of Transportation Statistics, National Transportation Statistics (NTS). Bureau of Transportation Statistics [Preprint] (2019). https://doi.org/10.21949/1503663.

85. Eric A. DeVuyst, Roger Sahs, What is the Economic Cost of a Bale of Hay? - Oklahoma State University (2024). https://extension.okstate.edu/fact-sheets/what-is-the-economic-cost-of-a-bale-of-hay.html.

86. Chad Hart, 2025 Estimated Costs of Crop Production in Iowa. (2025).

87. John J. Hanchar, Economics of Producing Industrial Hemp in New York State: Projected Costs and Returns, 2019 Budgets (2019). https://bpb-us-e1.wpmucdn.com/blogs.cornell.edu/dist/a/7491/files/2017/04/HancharJohn-economics-of-industrial-hemp-2019-analysis-update-2019_01_22-28o6gqp.pdf?utm_source=chatgpt.com.

88. D. J. Beerling, D. Z. Epihov, I. B. Kantola, M. D. Masters, T. Reershemius, N. J. Planavsky, C. T. Reinhard, J. S. Jordan, S. J. Thorne, J. Weber, M. Val Martin, R. P. Freckleton, S. E. Hartley, R.

H. James, C. R. Pearce, E. H. DeLucia, S. A. Banwart, Enhanced weathering in the US Corn Belt delivers carbon removal with agronomic benefits. *Proc. Natl. Acad. Sci.* **121**, e2319436121 (2024).

89. CDR.fyi, Current Market Prices (2024). https://www.cdr.fyi/.

90. U.S. EPA, Draft of Report on the Social Cost of Greenhouse Gases: Estimates Incorporating Recent Scientific Advances. (2022).
